# Endoplasmic reticulum aminopeptidase 2 regulates CD4^+^ T cells pyroptosis in rheumatoid arthritis

**DOI:** 10.1186/s13075-024-03271-3

**Published:** 2024-01-25

**Authors:** Jianhua Zhang, Hao Cai, Weiwei Sun, Weijie Wu, Yunyi Nan, Yingchen Ni, Xinyuan Wu, Minhao Chen, Hua Xu, Youhua Wang

**Affiliations:** Department of Orthopaedics, Affiliated Hospital of Nantong University, Nantong, Jiangsu China

**Keywords:** Pyroptosis, CD4^+^ T cells, ERAP2, Rheumatoid arthritis, Hedgehog signaling pathway

## Abstract

**Objective:**

Rheumatoid arthritis (RA) is a chronic, progressive autoimmune disease with a complex pathogenesis that has not yet been fully elucidated, and T-cell pyroptosis is an important pathogenetic factor in RA. This study aimed to investigate the role of endoplasmic reticulum aminopeptidase 2 (ERAP2) in the pyroptosis of CD4^+^ T cells in RA and the specific molecular mechanism.

**Methods:**

Peripheral venous blood was collected from human subjects, and CD4^+^ T cells were isolated and activated to measure the level of pyroptosis and ERAP2 expression. Pyroptosis levels were assessed using immunofluorescence, flow cytometry, qRT-PCR, and Western blotting. Changes in pyroptosis levels were observed upon knockdown or overexpression of ERAP2. To detect activated Caspase-1 in tissues, chimeric mice were engrafted with human synovial tissue and reconstituted with human CD4^+^ T cells. CD4 + T cells were treated with GLI1 antagonists and SMO receptor agonists to detect changes in pyroptosis levels.

**Results:**

CD4^+^ T cell levels undergoing pyroptosis were found to be elevated in the blood and synovium of RA patients. The gene and protein expression of ERAP2 were significantly higher in CD4^+^ T cells from RA patients. Deletion of ERAP2 suppressed pyroptosis of these cells, attenuated the activation of Caspase-1 in tissue T cells, and reduced tissue inflammatory responses. Reciprocally, overexpression of ERAP2 triggered inflammasome assembly, activated Caspase-1, and induced pyroptosis in CD4^+^ T cells. Mechanistically, ERAP2 inhibits the Hedgehog signaling pathway and upregulates the expression of nucleotide-binding oligomerization segment-like receptor family 3(NLRP3), cleaved Caspase-1, and Gasdermin D to promote pyroptosis in CD4^+^ T cells.

**Conclusions:**

Taken together, our results identify a novel mechanism by which ERAP2 regulates RA development and document the effect of the ERAP2/Hedgehog signaling axis on pyroptosis of CD4^+^ T cells from RA patients.

**Supplementary Information:**

The online version contains supplementary material available at 10.1186/s13075-024-03271-3.

## Introduction

Rheumatoid arthritis (RA) is a chronic autoimmune inflammatory disease that involves inflammation of the synovial joints and causes the progressive destruction of cartilage and bone, leading to joint deformities [1, 2]. The development of RA is influenced by multiple risk factors, including environmental and genetic variables, and is characterized by the production of excessive inflammatory mediators such as cytokines, chemokines, and autoantibodies associated with excessive activation of auto-reactive T and B cells which mediate autoimmune responses in the joints [3, 4]. Of these, CD4^+^ T lymphocytes are crucial for RA pathogenesis. Recent studies have shown that the percentage of activated Caspase-1 in CD4 + T cells from RA patients is significantly higher than in normal controls, and that inhibition of the DNA repair nuclease MRE11A can lead to mitochondrial dysfunction of CD4 + T cells, resulting in NLRP3 inflammasome assembly, Caspase-1 activation and Pyroptosis of RA CD4 + T cells [5].

Pyroptosis is a type of programmed cell death that contributes to inflammation; as the assembly of inflammatory vesicles proceeds, GSDMD is cleaved at the GSDMD-N terminus, characterized by the formation of pores in the cell membrane and the synthesis and release of pro-inflammatory cytokines, particularly IL-1β and IL-18 [6–8]. Recent studies have found that pyroptosis is involved in the occurrence and progression of RA, and large amounts of IL-1β and IL-18 are present in RA patients. In RA monocytes, complement C1q and Pentaxin 3 (PTX3) synergistically promote NLRP3 inflammasome over-activation and pyroptosis [9]. Succinate in synovial fibroblasts acts as a metabolic signal linking inflammation to fibrosis through activation of the NLRP3 inflammasome [10]. Acid-sensing ion channel 1a (ASIC1a) mediates chondrocyte pyroptosis in RA by promoting NLRP3 inflammatory vesicle assembly, Caspase-1 expression, and IL-1β and IL-18 release [11]. Therefore, studying the mechanism of pyroptosis in RA patients may help to identify new therapeutic targets. It was found that deletion of MRE11A led to significantly increased pyroptosis of CD4^+^ T cells in the synovium of a humanized mouse model of RA, and the expression of related inflammatory factors in the synovium was also significantly increased [5]. High levels of Caspase Recruitment Domain Family Member 8 (CARD8) were found to trigger a form of cell death in naïve and memory CD4^+^ T cells isolated from the peripheral blood of normal individuals, reminiscent of pyroptosis according to its morphological features [12]. In a study of systemic lupus erythematosus (SLE), significant cell pyroptosis was found in CD4^+^ T cells from patients and SLE model mouse kidneys. hsa_circ_4 regulates CD4^+^ T cell pyroptosis in SLE patients through the miR-125a-3p/GSDMD axis [13]. In parallel, CD4^+^ T cells from RA patients showed significant metabolic changes, with both naïve and memory T cells exhibiting premature aging, DNA breaks, accelerated telomere loss, dysregulation of G2/M cell cycle checkpoints, and continued differentiation to Th1 and Th17 effector cells [14, 15]. Therefore, it would be useful to investigate the relationships between RA, pyroptosis, and CD4^+^ T cells to open up new mechanistic avenues to treat RA. In the potential therapies for RA, glucocorticoids remain the mainstay of treatment, although one-third of patients gain no significant therapeutic benefit from their use. A recent study seeking potential predictors of clinical response showed that gene expression profiling of CD4^+^ T cells and monocytes from RA patients before pulse therapy with prednisolone revealed high expression of Endoplasmic Reticulum Aminopeptidase 2 (ERAP2) by these important players in the pathogenesis of RA [16]. ERAP2 is a key enzyme for the generation of antigenic epitopes that bind to major histocompatibility complex class I (MHC-I) [17] and has been shown to be a risk factor for three MHC-I associated diseases (birdshot chorioretinopathy [18], ankylosing spondylitis [19] and psoriasis [20]). ERAP2 regulates the immunopeptidome on the cell surface which triggers immune responses via T cells and is thus a potential target for treating autoimmune diseases, cancer, or viral infections [21]. As noted above, the role of ERAP2 in shaping immune responsiveness has been demonstrated in several autoimmune and inflammatory diseases [22]. Consistent with this, the addition of human recombinant ERAP2 to Peripheral blood mononuclear cells (PBMC) significantly increased the release of IL-1β and the expression of IL-18 and Caspase-1. Stimulation by human recombinant ERAP induced Apoptosis-associated speck-like protein containing CARD (ASC) molecular aggregation in the cytoplasm, which would lead to the activation of inflammasomes [23], a prerequisite for the occurrence of traditional pyroptosis. Therefore, whether ERAP2 can influence the development and progression of RA by regulating the level of CD4^+^ T pyroptosis in RA patients deserves further investigation.

In the present study, we found significant pyroptosis in CD4^+^ T cells from RA patients. ERAP2 expression was increased in these CD4^+^ T cells with an exacerbated level of pyroptosis. Moreover, we found that the Hedgehog signaling pathway was closely associated with the occurrence of ERAP2-induced pyroptosis in CD4^+^ T cells. We used the NOD/ShiLtJGpt-Prkdcem26Cd52Il2rgem26Cd22/Gpt (NCG) mouse model to examine correlations of ERAP2, Caspase-1 activation, and tissue inflammation. We found that the knockdown of ERAP2 reduced the hydrolysis of Caspase-1 protein and dampened tissue inflammation. The present study documents a novel mechanism by which ERAP2 is regulated in RA, providing an ideal therapeutic target for this disease.

## Materials and methods

### Patients and controls

Thirty patients with rheumatoid arthritis were recruited from the Affiliated Hospital of Nantong University between September 2021 and June 2022. All fulfilled the diagnostic criteria for RA and were positive for anti-CCP antibodies and rheumatoid factor. Non-neoplastic, non-infectious disease, gender-age matched non-rheumatoid arthritis patients were used as normal controls (NCs). Ten milliliters of peripheral blood from patients and controls were collected for this study. The study met the ethical requirements of the Affiliated Hospital of Nantong University, permission was obtained from the Ethics Committee of the Affiliated Hospital of Nantong University (2020-L136), and written informed consent was obtained from all participants.

### Sample collection and T cell purification and culture

Approximately 10 ml of human peripheral blood was obtained by venipuncture, rested in EDTA-K2 anticoagulation tubes, mixed with an equal volume of PBS, and slowly layered onto Lymphoprep (STEMCELL Technologies, Vancouver, BC, Canada, #07801) in SepMate tubes (STEMCELL Technologies, #86,450). CD4^+^ T cells were isolated from PBMCs using the human CD4^+^ T cell isolation kit (STEMCELL Technologies, # 17,952). To induce effector T cells, purified CD4^+^ T cells were cultured in ImmunoCul-XF T Cell Expansion Medium (STEMCELL Technologies, # 10,981) and supplemented with 25μL/mL human CD3/CD28 T Cell Activator (STEMCELL Technologies, # 10,971) and 10 ng/mL human recombinant IL-2 (STEMCELL Technologies, # 78,036).

### Transfection of T cells

Activated CD4^+^ T cells were transfected for 72 h using GFP-tagged lentivirus with ERAP2-specific shRNA (shERAP2) and GFP-tagged lentivirus with ERAP2 (LV-ERAP2) and negative control lentivirus from Gikai Genetics (Shanghai, China). The target sequence of shERAP2 was GCUUUCCCAGUAGCCACUATT. The transfection efficiency was improved using a suspension cell-specific virus transfection kit, and after successful transfection, cells were selected using 2 μg/mL puromycin (Beyotime, Shanghai, China). In specific cases, GANT58 (20 μm) or Purmorphamine (20 μm) from MedChemExpress (NJ, USA) were used to coculture with CD4^+^ T cells for 48 h.

### Immunofluorescence staining

Paraffin-embedded sections of human synovial tissue were deparaffinized and antigen repaired using standardized methods. The slides were incubated for 60 min using Immunol Staining Blocking Buffer (Beyotime, P0102), washed well with PBS, and incubated overnight at 4 °C with Immunol Staining Primary Antibody Dilution Buffer (Beyotime, P0103) prepared with anti-human Caspase-1 (1:50, 22,915–1-AP) and anti-human CD3 (1:300, 60,181–1-Ig) from Proteintech (Chicago, USA). After washing, slides were incubated for 1 h with a mixture of Alexa Fluor 647-labeled goat anti-rabbit IgG (H + L) (1:500, Beyotime, A0468) and Alexa Fluor 488-labeled goat anti-mouse IgG (H + L) (1:500, Beyotime, A0428) antibodies. The nuclei were stained with DAPI (Beyotime, P0131), and the stained tissues were examined under an Olympus microscope (Olympus, BX41).

### Western blotting

Protein was extracted from CD4^+^ T cells using the Minute Total Protein Extraction Kit for Animal Cultured Cells (SD-001, Invent Biotechnologies, MN, USA). The buffer contains a 1% mixture of protease and phosphatase inhibitors. The proteins were transferred to PVDF membranes by electrophoresis using SDS-PAGE gels (Epizyme, Shanghai, China). Blocking treatment was performed using NcmBlot Blocking Buffer. Antibodies were against ASC/TMS1 (Proteintech, 10,500- 1-AP), NLRP3 (Proteintech, 19,771–1-AP), GLI1 (Proteintech, 66,905–1-Ig), Caspase-1 (ab207802) and ERAP2 (Abcam, ab69037) from Abcam (Cambridge, USA), GSDMD (NBP2-33,422) from Novus (CO, USA), SHH (sc-166685) and SMO (Santa Cruz, sc-365112) from Santa Cruz (TX, USA). HRP-labeled goat anti-rabbit IgG (H + L) (Beyotime, A0208) and HRP-labeled goat anti-mouse IgG (H + L) (Beyotime, A0216) were used as secondary antibodies. Anti-beta-actin (Proteintech, 66,009–1-Ig) was used as the gel-loading control. The membranes were incubated with Abs, and then Western ECL (P10300) from NCM Biotech (Jiangsu China) was added for imaging with the Bio-Rad image system. The gray value of each band was measured using ImageJ.

### Quantitative RT-PCR analysis

Total RNA was purified from CD4^+^T cell cultures using the RNA-Quick Purification Kit (RN001) from EScience (Shanghai, China), according to the manufacturer’s instructions. Reverse transcription to cDNA was performed by using HiScript III RT SuperMix for qPCR (R323) from Vazyme (Nanjing, China). Quantitative RT-PCR was performed using the ChamQ Universal SYBR qPCR Master Mix (Vazyme, Q711) on a LightCycler96 (Roche) according to the kit instructions. Primer sequences used to amplify *ERAP2*, *NLRP3*, *ASC*, *GSDMD*, *Caspase-1*, *IL6*, *IL10*, *IL1B*, *TGFB1*, and *TNFA* are given in Supplemental Table 1.

### Measurement of secreted IL-1β

CD4^+^ T cells isolated from normal individuals or RA patients were cultured with anti-CD3/CD28 beads, and supernatants were harvested for cytokine analysis. ELISA kits were used to measure levels of IL-1β (EK101B) from Multi sciences (Shanghai, China).

### Measurement of LDH

CD4^+^ T cells were cultured with anti-CD3/CD28 beads. The LDH assay was performed according to the manufacturer’s instructions (C20300) from Invitrogen, (California, USA). Relative LDH release was calculated as LDH release [%] = 100 × (experimental sample LDH activity − spontaneous LDH release activity)/(maximum LDH release activity − spontaneous LDH release activity).

### Assessment of activated effector Caspases-1

Activated Caspase-1 was detected with the Pyroptosis/Caspase-1 Assay Kit (#9158) from ImmunoChemistry Technologies (CA, USA) according to the manufacturer’s instructions. CD4^+^ T cells were incubated with FLICA solution at 37 °C for 1 h. CD4^+^ T cells were then incubated with FITC anti-human CD4 antibody (357,406) from BioLegend (CA, USA) for 30 min. Fluorescently labeled cells were immediately detected by flow cytometry (Agilent, Novocyte 2060R).

### 7AAD^+^ Annexin V^+^ cell detection

The Annexin V-PE/7-AAD Apoptosis Detection Kit (GA1018) from KeyGEN (Jiangsu, China) was used to detect cell death according to the manufacturer’s instructions. In general, cells were harvested and incubated with the 7-AAD solution and 1 µL of Annexin V-PE at room temperature. After staining, the cells were immediately analyzed by flow cytometry (Agilent, Novocyte 2060R).

### Hoechst 33,342/PI fluorescence staining

Hoechst 33,342 and PI double staining were used to identify cell death. CD4^+^ T cells were washed and centrifuged, and an Antifade Mounting Medium with Hoechst 33,342 and propidium iodide (Beyotime, P0137) was added to the cell suspension and examined under the microscope.

### Synovitis induction in chimeric mice

Chimeric mice engrafted with human synovial tissue and reconstituted with human CD4^+^ T cells were generated as previously described. Pieces of human synovium were transplanted into a subcutaneous pocket on the dorsal midline of NOD/ShiLtJGpt-Prkdcem26Cd52Il2rgem26Cd22/Gpt (NCG) mice (15 male mice, 10 to 14 weeks old; GemPharmatech). synovial tissue was harvested from the knee joints of patients undergoing lower extremity amputation due to trauma. Following engraftment, ERAP2 was knocked down by transfecting CD4^+^ T cells with either reference shRNA or ERAP2-specific shRNA. Ten million transfected cells were adoptively transferred into the mice. After 7 days, mice were sacrificed and synovial grafts were taken for immunofluorescence staining or RT-PCR tissue transcriptome analysis.

All animals were housed in a standardized animal facility at Nantong University. The animal experimental protocols were approved by the ethics committee of Nantong University. During the experimental period, animals were housed at the Nantong University Animal Center at a standard rate of 5 animals per cage, under suitable conditions of humidity and moisture, with free access to drinking water and food. Isoflurane was used for animal anesthesia.

### Statistical analysis

All data are expressed as mean ± SD. Statistical analysis was performed using a t-test, one-way ANOVA. *P* values < 0.05 were considered statistically significant. Statistical analysis of experimental data was performed using GraphPad Prism 8.

## Results

### Participants

The study enrolled 30 RA patients and 30 normal controls (NCs) from 2021 through 2022. We selected non-RA patients without arthritis as the controls. All controls had normal ESR and CRP at enrolment. The majority of the patients and controls were women (22/30 and 21/30). All of the patients and controls are Asian. The mean age of the RA patients and controls was 58.83 years (range 43–78 years) and 56.60 years (range 47–67 years) (Table 1).

**Table 1:** Baseline clinical and demographic characteristics of the participants

	NC population	RA population
Individuals (*n*)	30	30
Females (%)	70	73.3
Age at visit	56.6 ± 6.05	58.83 ± 8.74
Disease parameters		
Disease duration (years)	/	10.62 ± 8.99
ESR (mm/h)	7.67 ± 4.36	60.57 ± 26.85
CRP (mg/L)	2.32 ± 1.95	37.22 ± 27.59
Positive for RF (%)	/	100
Positive for CCP (%)	/	100
Morning stiffness (min)	/	15.2 ± 22.58
Medications (%)		
Corticosteroids	/	56.7
NSAIDs	/	26.7
csDMARDs	/	53.3
b/tsDMARDs	/	3.3
Traditional Chinese drugs	/	23.3

Data presented as means ± SD or relative frequency (%)

*NC* Normal controls,* RA* Rheumatoid arthritis, *ESR* Erythrocyte sedimentation rate, *CRP* C-reactive protein, *RF* Rheumatoid factor, *CCP* Cyclic citrullinated peptide antibody, *NSAIDs* Non-steroidal anti-inflammatory drugs, *csDMARDs* Conventional synthetic disease-modifying antirheumatic drugs, *b/tsDMARDs* Biological or targeted synthetic disease-modifying antirheumatic drugs.

The threshold value was used as the measured value when the response fell outside of the range of measurement.

### Pyroptosis of CD4^+^ T cells is increased in RA patients

**Fig. 1:**
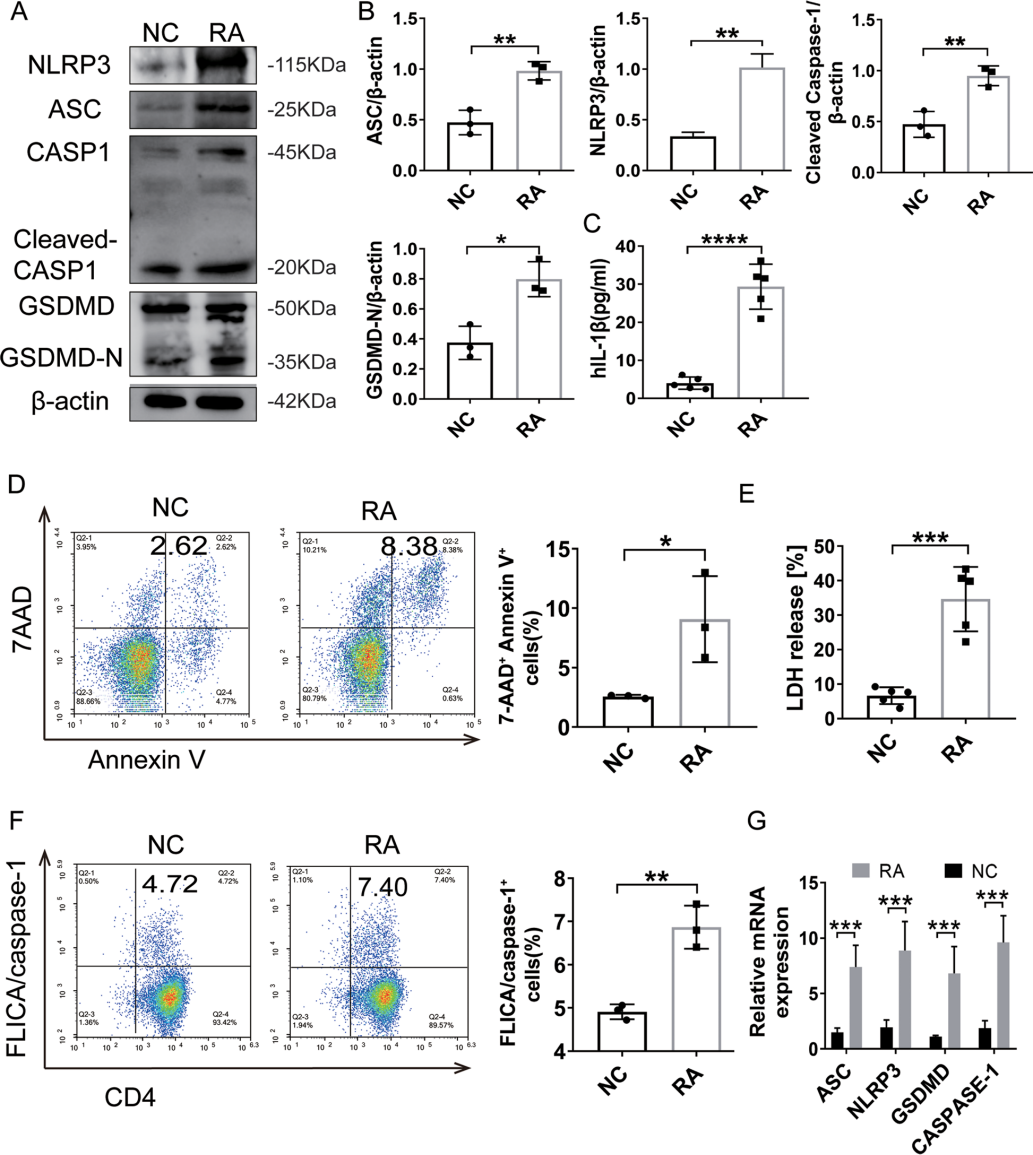
Pyroptosis of CD4^+^ T cells is increased in RA patients. **A** Representative immunoblots showing ASC, NLRP3, cleaved Caspase-1, and GSDMD-N expression in CD4^+^ T cells from NCs and RA patients. **B** Quantification of ASC, NLRP3, cleaved Caspase-1, and GSDMD-N protein expression shown in **A** (*n* = 3). **C** Concentrations of secreted IL-1β measured by ELISAs (*n* = 5). **D** CD4^+^ T cell death was quantified for control and RA CD4^+^ T cells on day 3 after stimulation by flow cytometric analysis of Annexin V^+^ 7AAD^+^ CD4^+^ T cells (*n* = 5). Representative flow plots of Annexin V and 7AAD staining of CD4^+^T cells and a histogram of the percentage of Annexin V^+^ 7AAD^+^ CD4^+^T cells are shown. **E** CD4^+^ T cell death was quantified by measuring LDH release (*n* = 5). **F** Cleaved Caspase-1 was identified by FLICA staining and flow cytometric analysis. Representative scatterplots of FLICA^+^ CD4^+^ T cells in the controls and RA patients are shown. **G** NLRP3, Caspase-1, GSDMD, and ASC transcripts compared in NCs and RA CD4 + T cells. All data are presented as the mean ± SD. Statistical significance was determined by Student’s *t* test; *p < 0.05, **p < 0.01, ***p < 0.001, ****p < 0.0001. NC, normal control; RA, rheumatoid arthritis; ASC, apoptosis-associated speck-like protein containing a CARD; NLRP3: NOD-like receptor protein 3; CASP1, Caspase-1; GSDMD-N, N-terminal pore-forming domain of GSDMD

To investigate the pyroptosis of CD4^+^ T cells, we compared the pyroptosis levels of CD4^+^ T cells from RA patients and normal controls in both activated and resting conditions. In the resting state, both RA and NC CD4^+^ T cells exhibited low levels of Caspase-1 activation. However, as activation time passed, the difference in Caspase-1 activation levels between the two groups became increasingly apparent. At 72 h, a significant difference in active Caspase-1 levels was observed between RA and NC (Supplemental Fig. 1). So, we isolated CD4^+^ T cells and extracted the related proteins after culturing the cells for 72 h. We assessed the expression of ASC, NLRP3, cleaved Caspase-1, and GSDMD-N. We found that the levels of these pyroptosis-related proteins were significantly higher in RA patients than in controls (Fig. 1A, B). Flow cytometry indicated a 2–threefold higher death rate of CD4^+^ T cells in RA patients than NCs (Fig. 1D). In the classical signaling pathway, activation of the inflammasome by the corresponding ligand promotes the self-cleavage of the precursor Caspase-1 to yield activated Caspase-1. To investigate the activation of Caspase-1 in RA CD4^+^ T cells, the cells were stained using the Caspase-1 detection kit. This revealed that the proportion of activated Caspase-1 in RA CD4^+^ T cells was 1.5 times that in NCs (Fig. 1F). We also assessed the release levels of LDH and IL-1β in CD4^+^ T cell cultures from RA patients, and found that levels were significantly higher in RA than in NCs (Fig. 1C, E). Thus, we documented the presence of significant lysis and large amounts of activated Caspase-1 in RA CD4 + T cells. *NLRP3*, *ASC*, *Caspase-1*, and *GSDMD* gene expression was significantly elevated (Fig. 1G), consistent with the protein levels. In conclusion, we postulated that CD4^+^ T cell pyroptosis may be closely related to the development of RA.

### ERAP2 regulates pyroptosis in CD4^+^ T cells

**Fig. 2:**
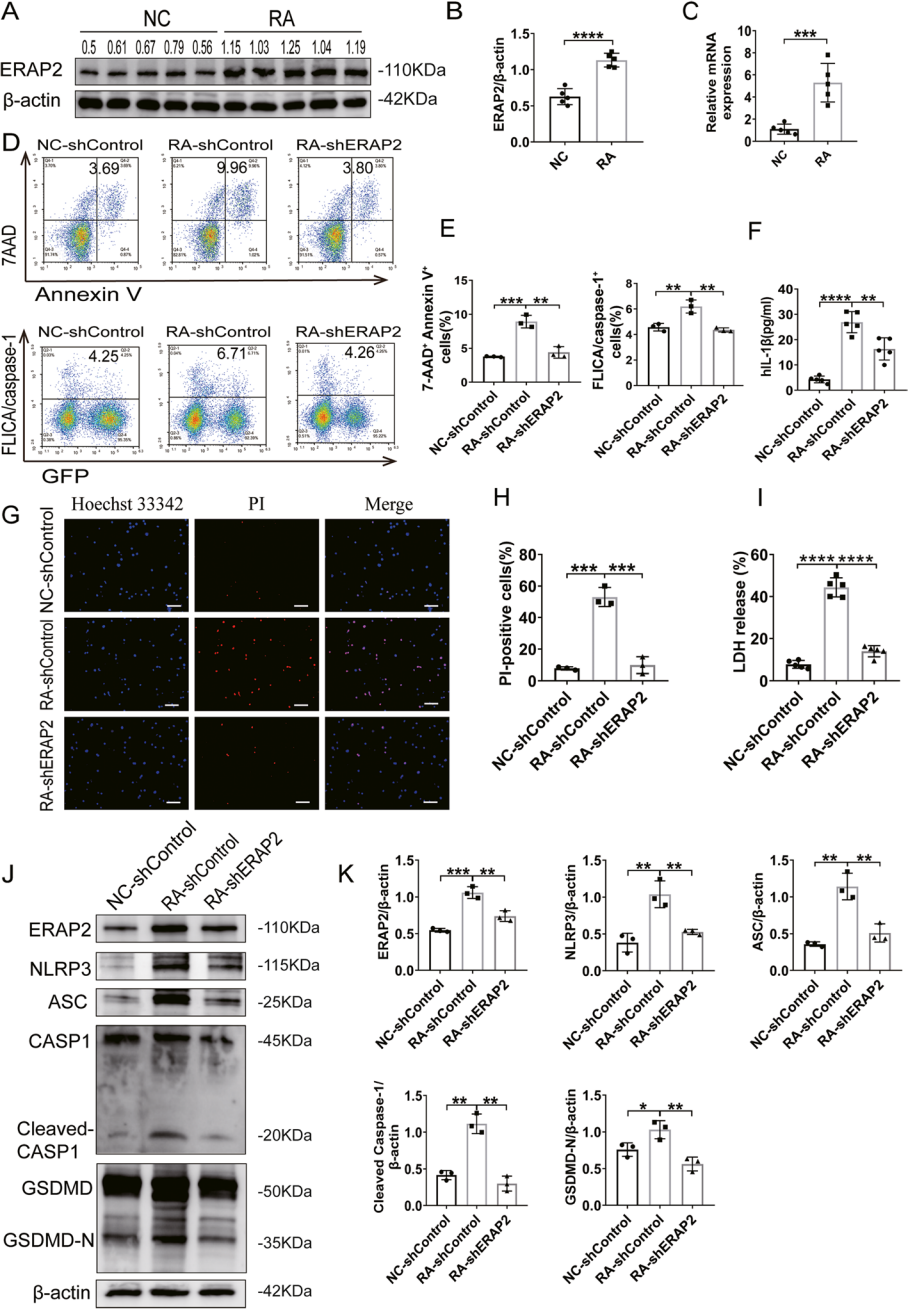
ERAP2 regulates pyroptosis in CD4^+^ T cells. NC and RA CD4^+^ T cells were stimulated for 72 h. Then, NC CD4^+^ T cells were infected with control shRNA virus (NC-shcontrol), and RA CD4^+^ T cells were infected with control shRNA virus or ERAP2 shRNA virus (RA-shcontrol/ RA-shERAP2). **A**, **B** ERAP2 expression in isolated CD4^+^ T cells is shown in representative immunoblots (left) and plots of the relative band density for NCs (*n* = 5) and RA patients (*n* = 5) with normalization to β-actin expression (right). **C** ERAP2 transcript levels in NC and RA CD4^+^ T cells (*n* = 5). **D**, **E** Flow cytometric analysis of CD4^+^ T cells treated as indicated and stained with Annexin V/7AAD (*n* = 3). Representative flow plots of Annexin V and 7AAD staining of CD4^+^ T cells and a histogram of the percentage of Annexin V^+^ 7AAD^+^ CD4^+^ T cells are shown, and representative scatterplots of FLICA^+^ cells identified by flow cytometric analysis (*n* = 3). **F** Concentrations of secreted IL-1β measured by ELISAs (*n* = 5). **G**, **H** Pyroptotic cells were identified by Hoechst 33,342/PI staining; the nuclei were stained blue with Hoechst 33,342, while pyroptotic cells were stained red with PI (*n* = 3). Scale bars: 50 μm. **I** T-cell death was quantified by measuring LDH release (*n* = 5). **J** Representative immunoblots showing ASC, NLRP3, cleaved Caspase-1, and GSDMD-N expression (*n* = 3). **K** Quantification of ASC, NLRP3, cleaved Caspase-1, and GSDMD-N protein expression shown in **J** (*n* = 3). All data are shown as the mean ± SD. Statistical significance was determined by one-way ANOVA followed by Bonferroni’s multiple-comparisons test for multigroup comparisons or Student’s *t* test; *p < 0.05, **p < 0.01, ***p < 0.001, ****p < 0.0001. NC, normal control; RA, rheumatoid arthritis

ERAP2 is closely associated with autoimmune diseases and cell pyroptosis. To investigate the specific mechanism of pyroptosis in RA CD4^+^ T cells, we analyzed their ERAP2 expression. This showed that the ERAP2 protein levels and gene expression were significantly higher in RA than in controls (Fig. 2A–C). These results suggest that elevated ERAP2 expression may be closely related to the pyroptosis of CD4^+^ T cells. To further investigate the specific role of ERAP2 in RA CD4^+^ T cell pyroptosis, we showed that knocking it down resulted in a 2–threefold reduction of cell death relative to the controls, accompanied by a significant decrease in the frequency of activated Caspase-1-positive cells (Fig. 2D, E). When cells undergo pyroptosis, activated GSDMD-N forms pores in the cell membrane, disrupting its integrity and causing cell death. To further verify that ERAP2 exacerbates cell death, we used Hoechst 33,342/PI fluorescent staining. This showed that the percentage of positive cells was much higher in RA CD4^+^ T cells than in controls and that the amount of PI uptake was significantly decreased after knockdown of ERAP2 (Fig. 2G, H). The levels of LDH and IL-1β release into the culture medium of CD4^+^ T cells from RA patients were decreased after ERAP2-knockdown by approximately 20% and 40%, respectively, relative to controls (Fig. 2F, I). To further confirm that ERAP2 affects the occurrence of pyroptosis in RA CD4^+^ T cells, we examined the expression of pyroptosis-related proteins. ASC, NLRP3, activated Caspase-1 and GSDMD-N were all significantly reduced on ERAP2 knockdown (Fig. 2J, K). Together, these findings suggest that ERAP2 plays a crucial role in the occurrence of CD4^+^ T cell pyroptosis in RA.

### Overexpression of ERAP2 promotes CD4^+^ T cell pyroptosis

**Fig. 3:**
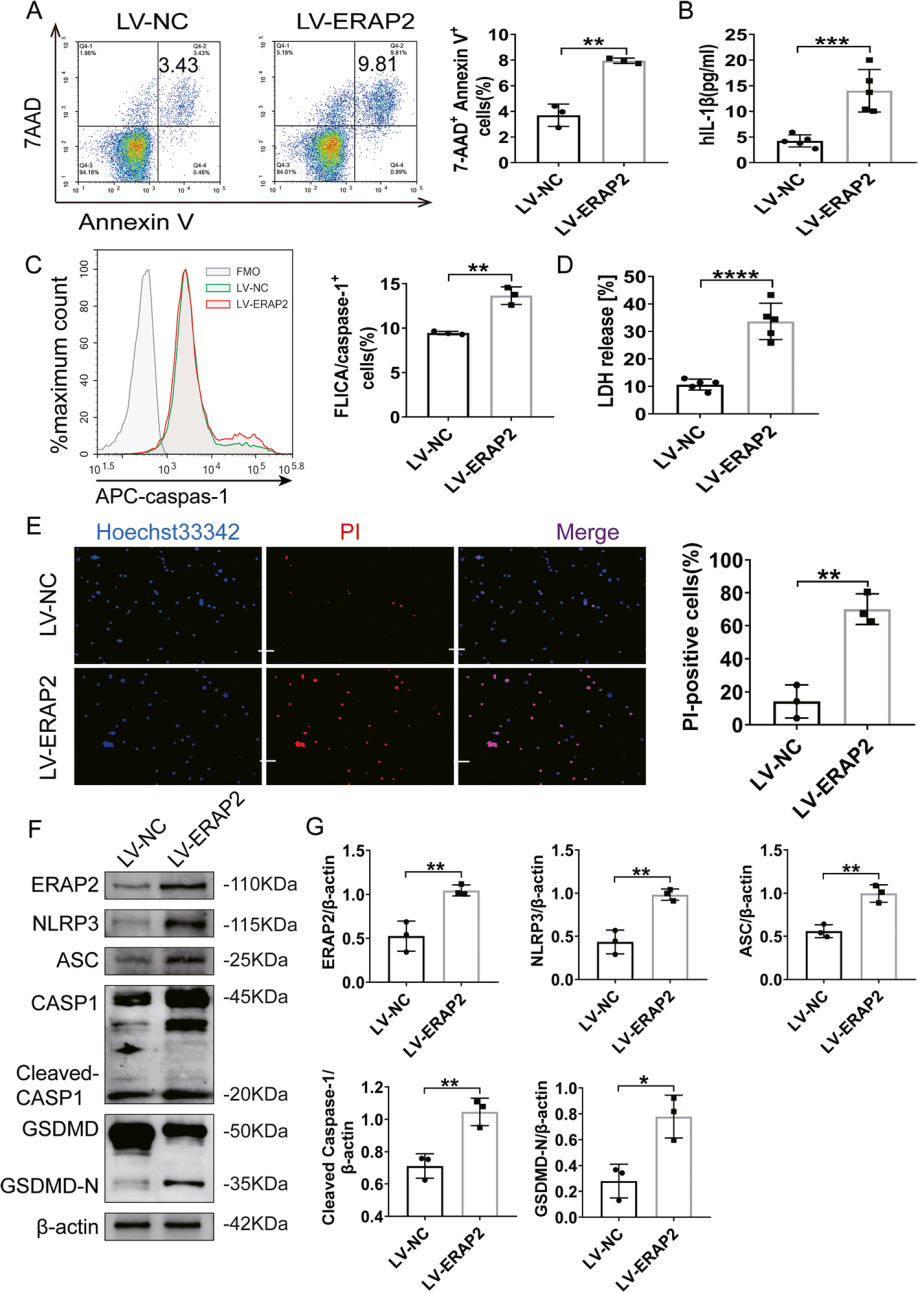
Overexpression of ERAP2 promotes CD4^+^ T cell pyroptosis. Normal CD4^+^ T cells were stimulated for 72 h. Then, CD4^+^ T cells were infected with control lentivirus or lentivirus encoding ERAP2 (LV-NC/ LV-ERAP2). **A** Flow cytometric analysis of CD4^+^ T cells treated as indicated and stained with Annexin V/7AAD (*n* = 3). Representative flow plots of Annexin V and 7-AAD staining of CD4^+^T cells and a histogram of the percentage of Annexin V^+^ 7AAD^+^ CD4^+^T cells are shown. **B** Concentrations of secreted IL-1β measured by ELISAs (*n* = 5). **C** Representative scatterplots of FLICA^+^ cells identified by flow cytometric analysis (*n* = 3). **D** T-cell death was quantified by measuring LDH release (*n* = 5). **E** Pyroptotic cells were identified by Hoechst 33,342/PI staining; the nuclei were stained blue with Hoechst 33,342, while pyroptotic cells were stained red with PI (*n* = 3). Scale bars: 50 μm. **F** Representative immunoblots showing ASC, NLRP3, cleaved Caspase-1, and GSDMD-N expression (*n* = 3). **G** Quantification of ASC, NLRP3, cleaved Caspase-1, and GSDMD-N protein expression shown in **F** (*n* = 3). All data are presented as the mean ± SD. Statistical significance was determined by Student’s *t* test; *p < 0.05, **p < 0.01, ***p < 0.001, ****p < 0.0001. NC, normal control; RA, rheumatoid arthritis

To further investigate the role of ERAP2 in CD4^+^ T cell pyroptosis, we treated NC CD4^+^ T lymphocytes with a lentivirus encoding ERAP2 (LV-ERAP2). Notably, overexpression of ERAP2 significantly induced CD4^+^ T pyroptosis, with a twofold increase in mortality compared to controls (Fig. 3A). Flow cytometric results showed that activated Caspase-1-positive CD4^+^ T cells were significantly increased on overexpression of ERAP2 (Fig. 3C). Levels of LDH and IL-1β showed the same upward trend (Fig. 3B, D) as did the Hoechst 33,342/PI staining (Fig. 3E). Uptake of PI by CD4^+^ T cells was significantly upregulated after overexpression of ERAP2, suggesting that the integrity of the membrane of a large number of CD4^+^ T cells was disrupted. We found that ERAP2 protein was significantly increased in CD4^+^ T cells after overexpression of ERAP2, while the expression of inflammasome ASC and NLRP3 was also significantly upregulated, and cleaved Caspase-1 and GSDMD-N were twofold upregulated relative to controls (Fig. 3F, G). Together, these results suggest that the overexpression of ERAP2 exacerbates CD4^+^ T cell pyroptosis.

### ERAP2 regulates Caspase-1 activation in synovial T cells

**Fig. 4:**
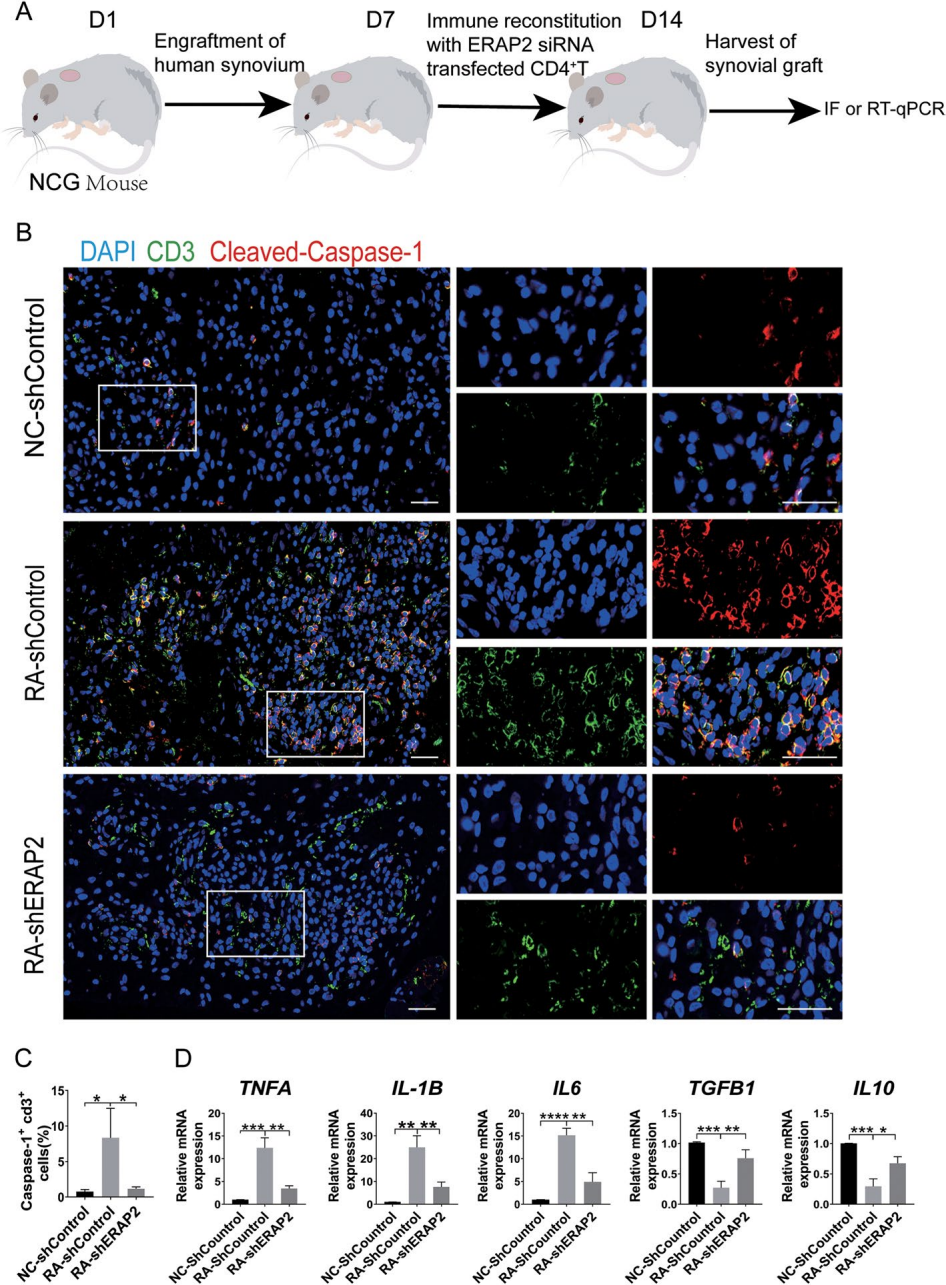
ERAP2 regulates Caspase-1 activation in synovial T cells. **A** NCG mice engrafted with human synovial tissue were reconstituted with CD4^+^ T cells from controls or RA patients and ERAP2 was silenced in CD4^+^ T cells before transfer. The synovial grafts were explanted for imaging analysis. The illustrations were created with Figdraw.com. **B** Immunofluorescence staining of cleaved Caspase-1 (red) and CD3 (green) in synovial tissue harvested from humanized NCG mice. The right panels show higher-magnification views of the boxed areas in the left panels. Scale bars: 50 μm. **C** Percentages of CD3 + cleaved Caspase-1 + T cells (*n* = 3). **D** Tissue transcriptome analysis of inflammation-related genes by RT‒qPCR (*n* = 3). TNFA, IL6 and IL1B are proinflammatory genes. IL10 and TGFB1 are anti-inflammatory genes. All data are the mean ± SD. Statistical significance was determined by one-way ANOVA followed by Bonferroni’s multiple-comparisons test for multigroup comparisons; *p < 0.05, **p < 0.01, ***p < 0.001, ****p < 0.0001. NC, normal control; RA, rheumatoid arthritis

To further investigate how ERAP2 regulates CD4^+^ T cell pyroptosis in RA, we extended these findings to synovial tissue in vivo. We established a humanized NCG mouse model, and to mimic ERAP2 low-expressing CD4^+^ T cells, RA CD4^+^ T cells were infected with shERAP2 before tail vein injection. Synovium was removed 7 days thereafter (Fig. 4A). Immunofluorescence staining showed that knockdown of ERAP2 significantly reduced CD4^+^ T cell density in synovial tissues and that activated Caspase-1 in the CD4^+^ T cells was also significantly reduced (Fig. 4B, C). These results suggest that high expression of ERAP2 leads to marked activation of Caspase-1 in CD4^+^ T cells in the synovium, which contributes to pyroptosis, breaking tissue tolerance, and increasing levels of the intrinsic pro-inflammatory cytokines *TNF*, *IL6*, and *IL1B*. This leads to intense synovial tissue inflammation. In contrast, the expression of two anti-inflammatory molecules, *IL10* and *TGFB1*, was significantly downregulated but significantly upregulated again after knocking down ERAP2 (Fig. 4D). Collectively, these results link ERAP2 to triggering Caspase-1 activation in CD4^+^ T cells in tissues, suggesting that it is a key factor in the pyroptosis of CD4^+^ T cells in the synovium.

### ERAP2 induces pyroptosis in RA CD4^+^ T cells by inhibiting the Hedgehog signaling pathway

**Fig. 5:**
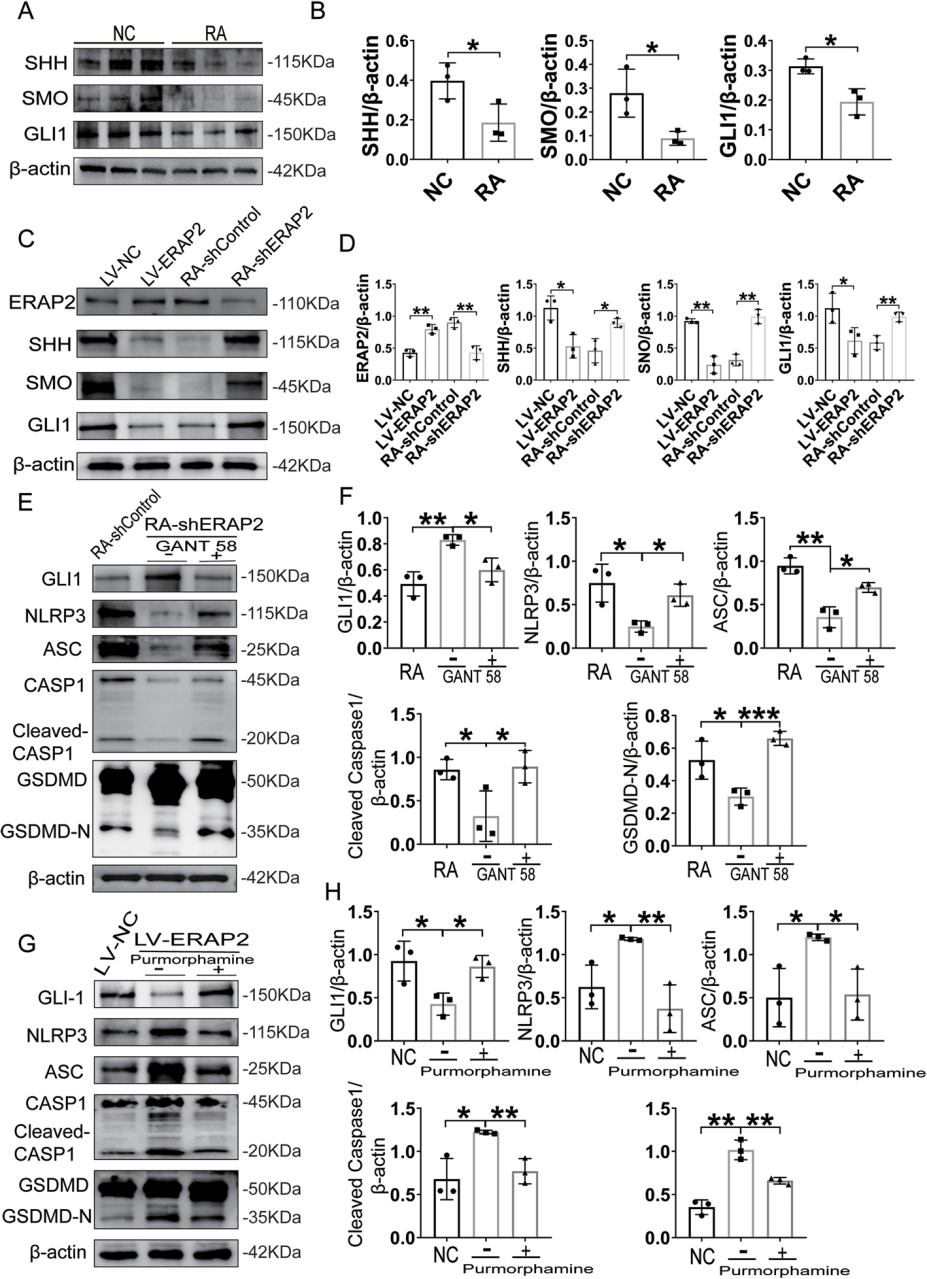
ERAP2 induces pyroptosis in RA CD4 + T cells by inhibiting the Hedgehog signaling pathway. CD4^+^ T cells were stimulated for 72 h. Then, normal CD4^+^ T cells were infected with control lentivirus or lentivirus encoding ERAP2 (LV-NC/ LV-ERAP2). RA CD4 + T cells were infected with control shRNA virus or ERAP2 shRNA virus (RA-shcontrol/ RA-shERAP2). **A**, **B** SHH, SMO, and GLI1 expression in isolated CD4 + T cells is shown in representative immunoblots (left) and plots of the relative band density for NCs (*n* = 3) and RA patients (*n* = 3) with normalization to β-actin expression (right). **C**, **D** ERAP2, SHH, SMO, and GLI1 expression in isolated CD4 + T cells with overexpression or knockdown is shown in representative immunoblots (left) and plots of the relative band density with normalization to β-actin expression (right). **E**, **F** GLI1, ASC, NLRP3, cleaved Caspase-1 and GSDMD-N expression in isolated CD4 + T cells with GANT58 treatment (20 µm) is shown in representative immunoblots (left) and plots of the relative band density with normalization to β-actin expression (right). **G**, **H** GLI1, ASC, NLRP3, cleaved Caspase-1 and GSDMD-N expression in isolated CD4 + T cells with Purmorphamine treatment (20 µm) is shown in representative immunoblots (left) and plots of the relative band density with normalization to β-actin expression (right). All data are shown as the mean ± SD. Statistical significance was determined by one-way ANOVA followed by Bonferroni’s multiple-comparisons test for multigroup comparisons or Student’s *t* test; *p < 0.05, **p < 0.01, ***p < 0.001, ****p < 0.0001. NC, normal control; RA, rheumatoid arthritis

Previous studies have shown that the activity of the Hedgehog signaling pathway in RA patients’ serum [24], synoviocytes [25], and chondrocytes [26] is closely associated with RA disease severity. Furthermore, activation of the Hedgehog signaling pathway leads to the inhibition of NLRP3, which in turn inhibits NLRP3-driven cellular pyroptosis [27, 28]. Thus, ERAP2 may promote CD4^+^ T pyroptosis in RA by inhibiting the Hedgehog/SMO/Gli1 signaling axis. To test this hypothesis, we first determined the presence of molecules critical to the Hedgehog signaling pathway at the protein level in CD4^+^ T cells. This revealed that SHH, SMO, and GLI1 were more weakly expressed in CD4^+^ T cells from RA patients than in controls, whereas ERAP2 levels were higher (Fig. 5A, B). Furthermore, we found that when we used a Hedgehog pathway activator or inhibitor, we significantly changed the pyroptosis level of CD4 + T cells (Supplemental Fig. 2A, B).

We then overexpressed or knocked down ERAP2 using lentivirus transfection and found that the amount of ERAP2 present determined the levels of SHH, SMO, and Gli1. Thus, molecules associated with the Hedgehog/SMO/Gli1 signaling axis were repressed when ERAP2 was overexpressed, while they were more highly expressed when ERAP2 was knocked down (Fig. 5C, D). The above results demonstrate that ERAP2 affects cellular functions by inhibiting the activation of the Hedgehog signaling pathway in CD4^+^ T cells.

**Fig. 6:**
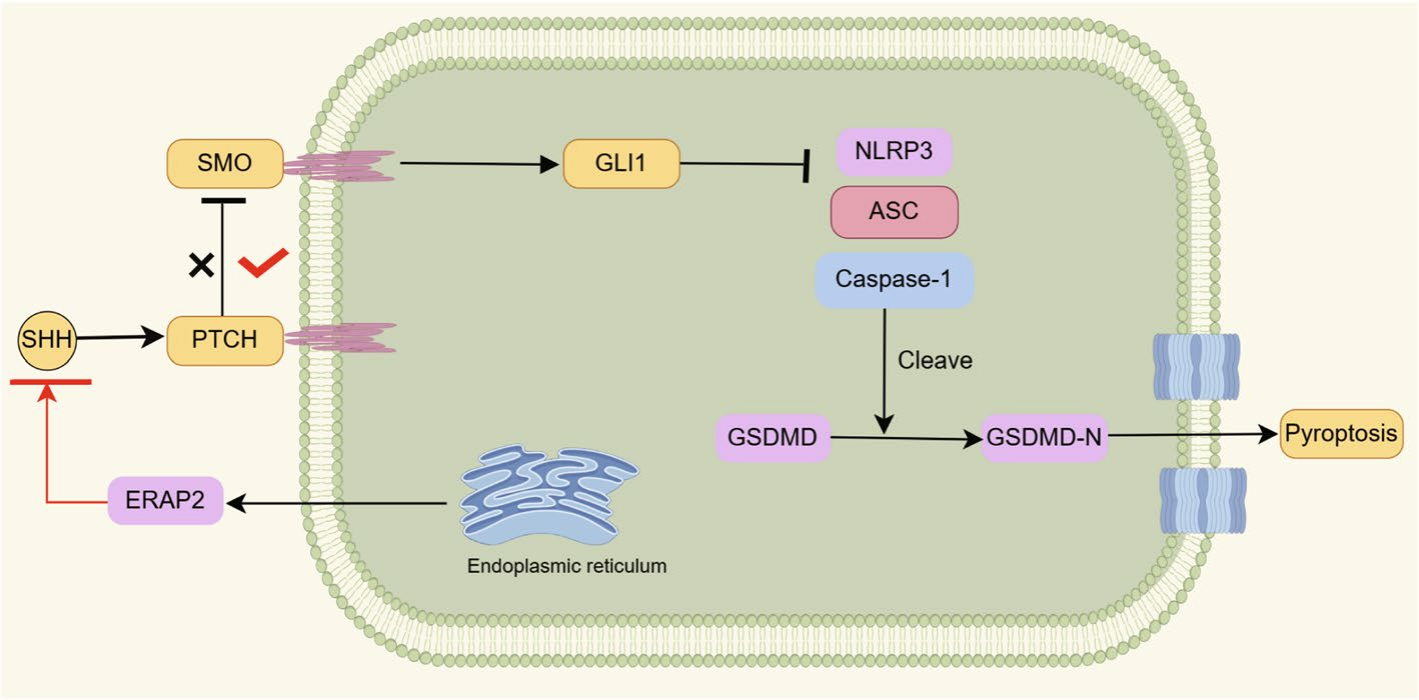
Schematic model illustrating the proposed mechanism of CD4 + T cell pyroptosis in RA

Because ERAP2 inhibits the activation of the Hedgehog signaling pathway, to further investigate the effect of its activation on CD4^+^ T cell pyroptosis, we used a novel GLI antagonist GANT58 and SMO receptor agonist Purmorphamine. Compared to controls, when ERAP2 was knocked down (Fig. 5E, F), GANT58 treatment decreased GLI1 expression and upregulated the expression of NLRP3, ASC, GSDMD-N, and activated Caspase-1 at the protein level in CD4^+^ T cells. In contrast, when ERAP2 was overexpressed (Fig. 5G, H), Purmorphamine treatment upregulated the expression of GLI1 but suppressed the increase of cell death due to ERAP2 overexpression. Taken together, these data suggest that the Hedgehog pathway is critical for the control of pyroptosis in CD4^+^ T cells in RA (Fig. 6).

RA CD4 + T cells overexpress ERAP2, and high levels of ERAP2 inhibit the SHH ligands, resulting in the inability of SHH ligands to bind to PTCH, which promotes the inhibitory effect of PTCH on SMO, which in turn leads to the loss of the inhibitory function of GLI1 on NLRP3. NLRP3 form an inflammasome complex followed by the recruitment of ASC and pro-Caspase-1. The inflammasome cleaves GSDMD to produce GSDMD-N, which induces pyroptosis in CD4 + T cells.

## Discussion

RA is a chronic inflammatory disease characterized by the infiltration of activated immune cells into the synovial membrane, leading to synovial hyperplasia, the formation of vascular opacities, and the progressive destruction of cartilage and bone. It has been suggested that RA is closely associated with cellular pyroptosis and apoptosis, but the underlying mechanisms are unclear [29]. Studies have shown that the development of RA is associated with inflammatory T cell infiltration and that T cells are the main inflammatory cell type that invades synovial tissue. Of these, CD4^+^ T cells are the main players involved in the pathological processes leading to RA [30]. Therefore, understanding the pathogenic mechanisms employed by such CD4^+^ T cells in RA is very important. ERAP2 is a key enzyme in the generation of antigenic epitopes on the cell surface which trigger immune responses, making them potential targets for autoimmune diseases [21, 31]. ERAP2 can increase the expression and release of IL-1β, IL-18, and Caspase-1, and also the aggregation of inflammatory vesicles in PBMCs of RA patients, which may eventually lead to the development of pyroptosis. However, to date, there are no studies on the effects of ERAP2 on CD4^+^ T cell pyroptosis in RA. In the present study, we demonstrated for the first time that ERAP2 promotes the pyroptosis of CD4^+^ T cells from RA patients and thus affects the development of the disease.

We isolated CD4^+^ T cells from the peripheral blood of RA patients and matched controls and quantified pyroptosis by various different methods. The results revealed increased LDH release, overactivation of Caspase-1, and high expression of GSDMD-N in CD4^+^ T cells from RA patients, indicating the occurrence of pyroptosis, the degree of which was significantly higher in patients than in controls. We also report that ERAP2 was involved in RA CD4^+^ T cell pyroptosis, with increased ERAP2 found at the gene transcription and protein levels. Pyroptosis of RA CD4^+^ T cells was increased or decreased after overexpression or knockdown of ERAP2, respectively. In addition, activation of Caspase-1 was significantly elevated in synovial transplanted mice, but could also be downregulated by knocking down ERAP2. These findings suggest that ERAP2-induced pyroptosis of CD4^+^ T cells may be a major promoter of RA.

ERAP2 is mainly located in the endoplasmic reticulum and is involved in the generation of antigens associated with MHC class I molecules. Thus, it plays a key role in the selection, regulation, and activation of specific T cell clones that can lead to enhanced or diminished immune responses. However, several studies have reported that in addition to acting enzymatically in the endoplasmic reticulum, ERAP2 may also be secreted into the extracellular environment where it mediates different pathophysiological functions [32]. There is growing evidence that proteins can have multiple functions depending on their cellular localization. ERAP2 can bind different substrates and exert physiological functions, thus playing different roles in the innate and acquired immune system as well as in the inflammatory response. Here, we document for the first time that ERAP2 leads to CD4^+^ T cell pyroptosis by inhibiting the Hedgehog signaling pathway.

The Hedgehog signaling pathway is an extremely conserved pathway in biological evolution, plays an important regulatory role in vertebrate embryonic development, cell proliferation, and differentiation, and is often inhibited in mature tissues. In the present study, we identified and demonstrated for the first time that the Hedgehog signaling pathway was significantly inhibited in RA CD4^+^ T cells, accompanied by a high level of cellular pyroptosis.

Moreover, when we activated or inhibited the Hedgehog signaling pathway using chemical reagents, the degree of pyroptosis of CD4^+^ T cells could be up- or downregulated. Recent studies have shown that Foxo1M-KO enhances the β-catenin-mediated activity of GLI1, a key molecule in the Hedgehog pathway, and also reduces receptor-interacting protein kinase 3 (RIPK3) and NIMA-related kinase 7 (NEK7)/NLRP3 expression, thereby attenuating tissue inflammatory responses [28]. In parallel, CD47-SIRPα activates the Hedgehog/SMO/Gli1 pathway, which controls NEK7/NLRP3 activity through interactions between Gli1 and NICD, thereby dampening NLRP3-driven inflammatory responses [27]. Indeed, increased Gli1 activity inhibits pro-inflammatory mediator production and tissue inflammation, whereas Gli1 deficiency promotes immune cell activation and inflammatory responses [33, 34]. These findings suggest that inhibition of the Hedgehog signaling pathway exacerbates RA CD4^+^ T cell pyroptosis.

It should be noted that although our study demonstrated that ERAP2 promotes CD4^+^ T cell pyroptosis in RA patients by inhibiting the Hedgehog signaling pathway, this role needs to be further validated in an in vivo animal model and an effective inhibitor of ERAP2 needs to be found to stop the development and progression of RA. In summary, our study showed that the development of RA is closely related to CD4^+^ T cell pyroptosis and that abnormal expression of ERAP2 is the main cause of such pyroptosis. Mechanistically, we found that ERAP2 promotes CD4^+^ T cell pyroptosis by inhibiting the Hedgehog signaling pathway. These results suggest that ERAP2 plays a key role in CD4^+^ T cell pyroptosis in RA and that targeting the ERAP2-Hedgehog signaling axis may represent a potential novel therapeutic strategy.

## Conclusions

In summary, our study found that ERAP2 was highly expressed in RA CD4^+^T cells and induced pyroptosis of CD4^+^T cells in RA by inhibiting Hedgehog signaling pathway. This study suggests that ERAP2 may be a potential therapeutic target for RA in the future.

### Supplementary Information

**Additional file 1. **

**Additional file 2: Supplemental Table 1. **Primer sequences for RT-qPCR. **Supplemental Figure 1.** Comparative analysis of caspase activation in control and RA CD4^+^ T cells. **Supplemental Figure 2. **A, B ERAP2 induces pyroptosis in RA CD4^+^ T cells by inhibiting the Hedgehog signaling pathway.

## Data Availability

All data are available within the article and supplementary files, or from the authors upon reasonable request.

## Abbreviations

RA: Rheumatoid arthritis
ERAP2: Endoplasmic reticulum aminopeptidase 2
NLRP3: Nucleotide-binding oligomerization segment-like receptor family 3
GSDMD: Gasdermin D
PTX3: Pentaxin 3
ASIC1a: Acid-sensing ion channel 1a
CARD8: Caspase Recruitment Domain Family Member 8
SLE: Systemic lupus erythematosus
MHC-I: Major histocompatibility complex class I
PBMC: Peripheral blood mononuclear cell
ASC: Apoptosis-associated speck-like protein containing CARD
NCG: NOD/ShiLtJGpt-Prkdcem26Cd52Il2rgem26Cd22/Gpt
NC: Normal controls
