# Supplementary material for: Endoplasmic reticulum aminopeptidase 2 regulates CD4+ T cells pyroptosis in rheumatoid arthritis
Source: Arthritis Res Ther. 2024 Jan 25;26:36. doi: 10.1186/s13075-024-03271-3 (PMC10810225; doi:10.1186/s13075-024-03271-3)
Supplement: Supplementary file 1 — Additional file 1. [file 13075_2024_3271_MOESM1_ESM.doc]

FIGURE 1 A


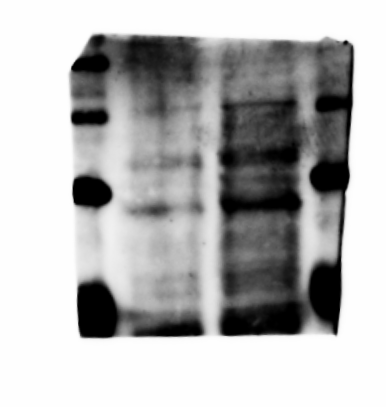


NLRP3


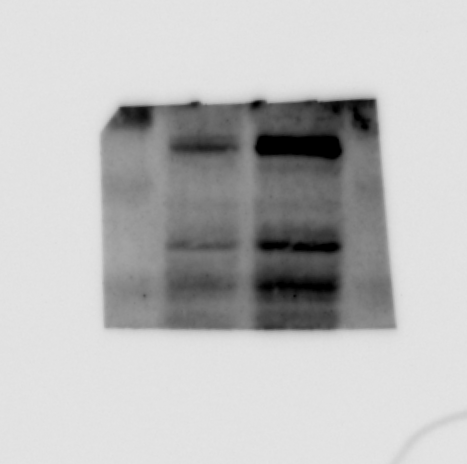


ASC


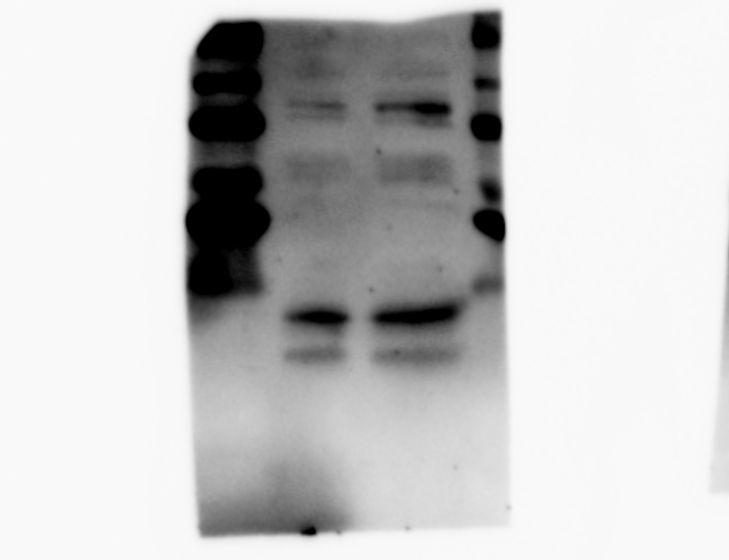


CASPASE-1


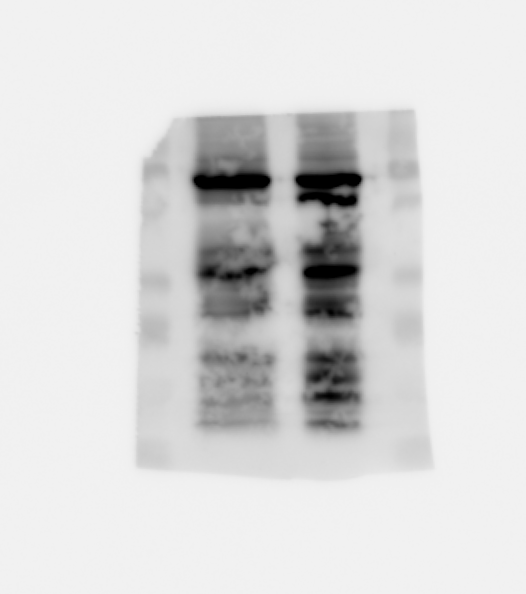


GSDMD


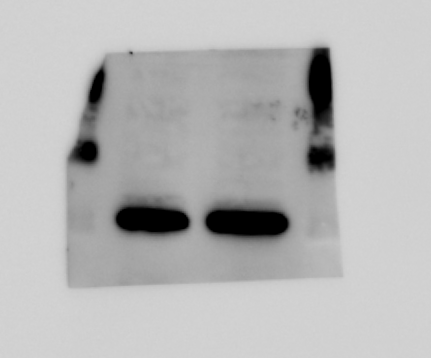


ACTIN

FIGURE 2 A


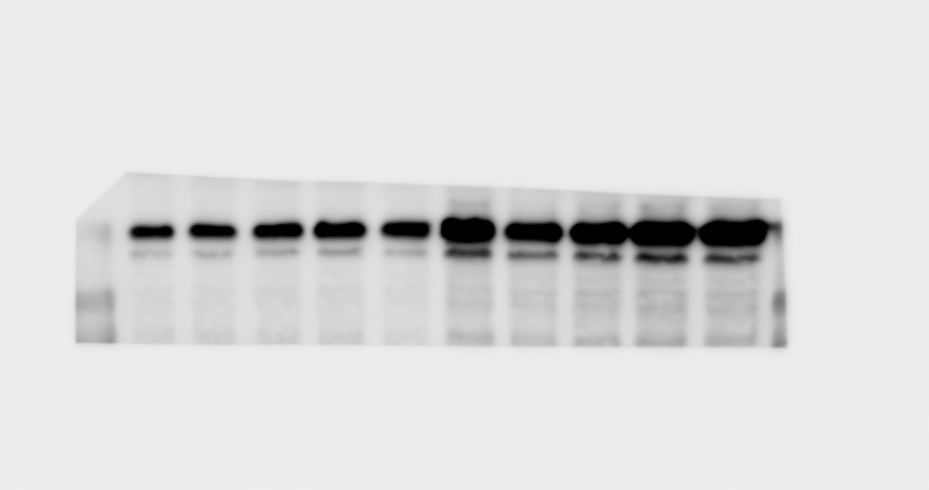


ERAP2


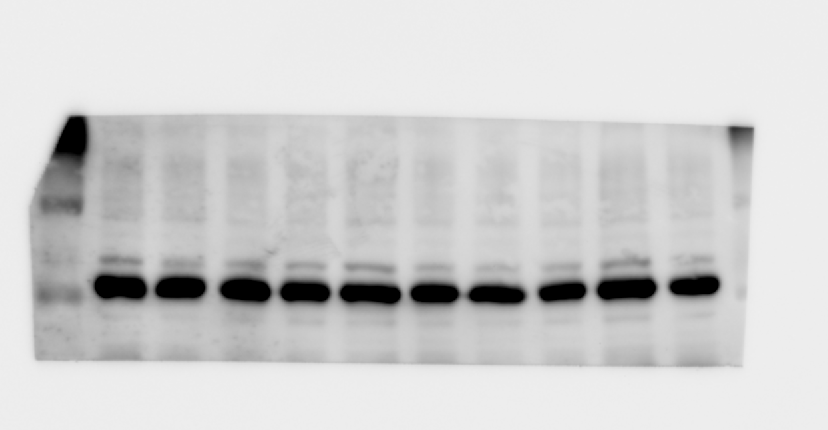


Actin


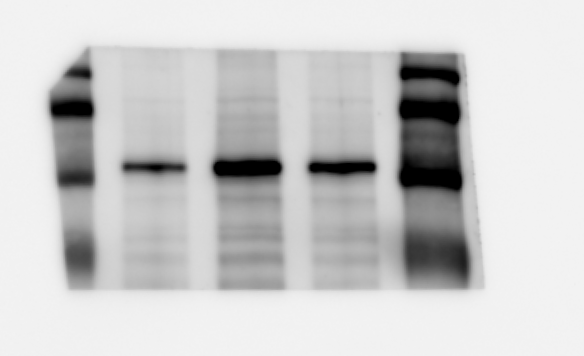
FIGURE 2G

ERAP2


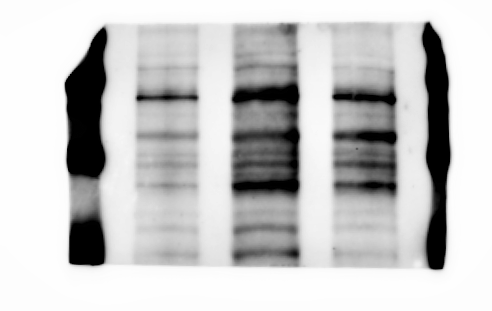


NLRP3


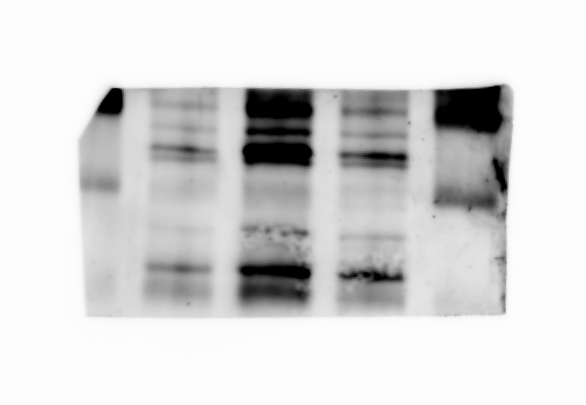


ASC


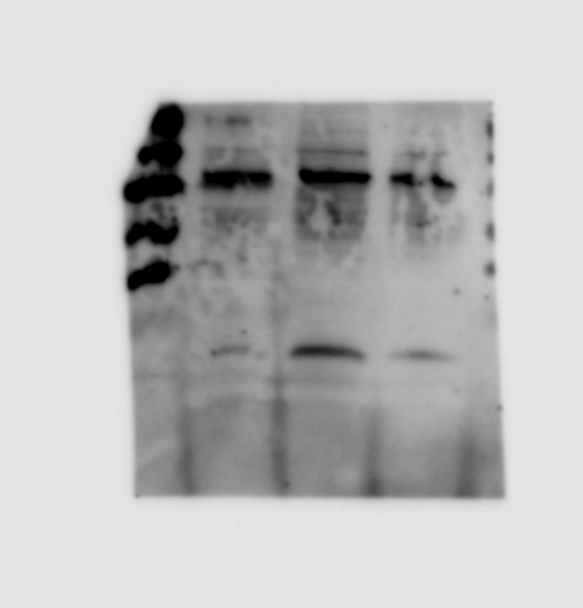


CASPASE-1


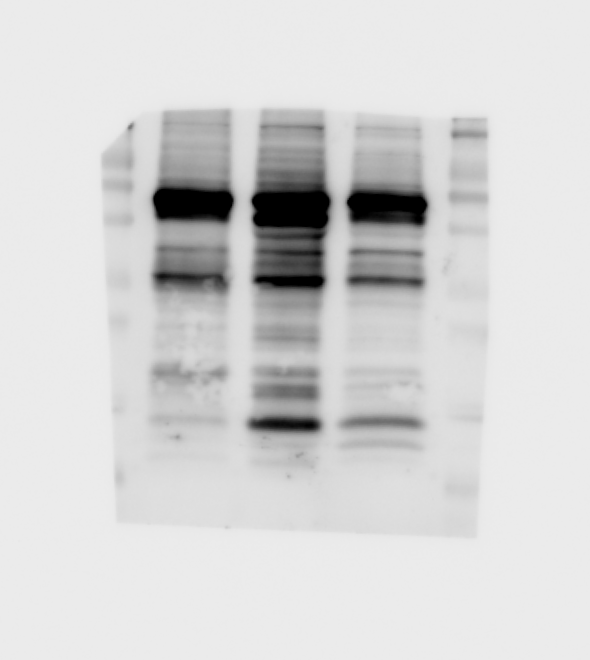


GSDMD


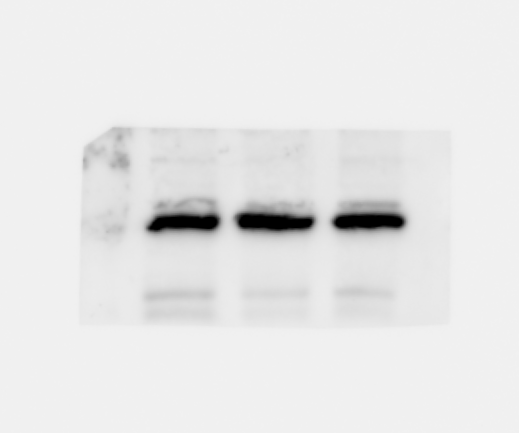


ACTIN

FIGURE 3 F


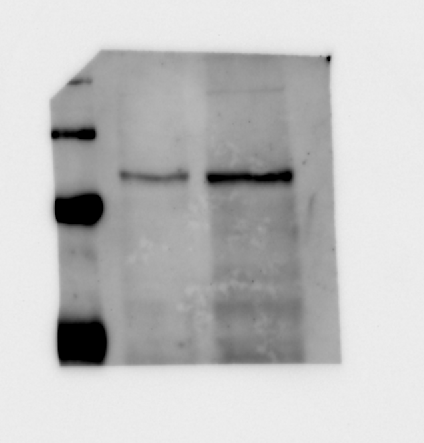


ERAP2


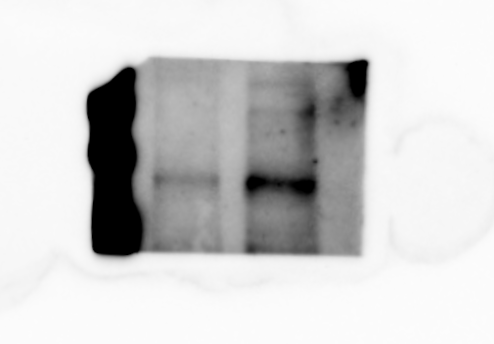


NLRP3


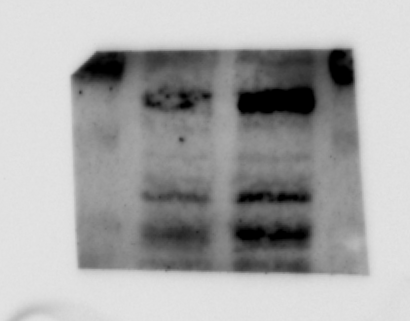


ASC


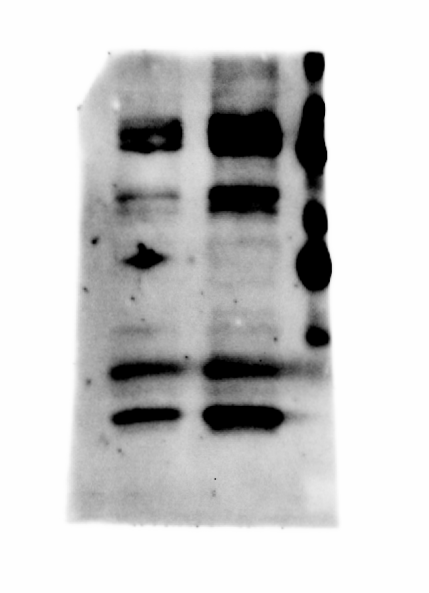


CASPASE-1


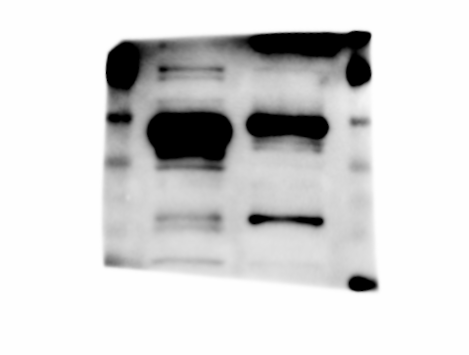


GSDMD


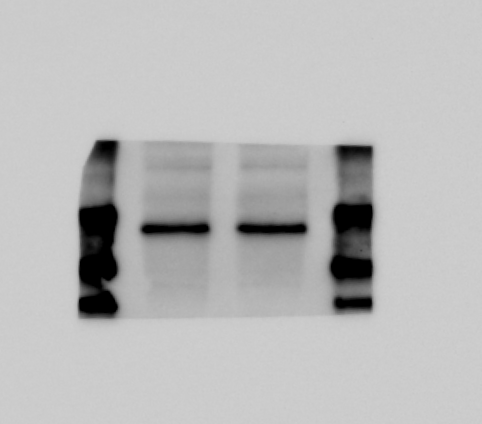


ACTIN

FIGURE 5 A


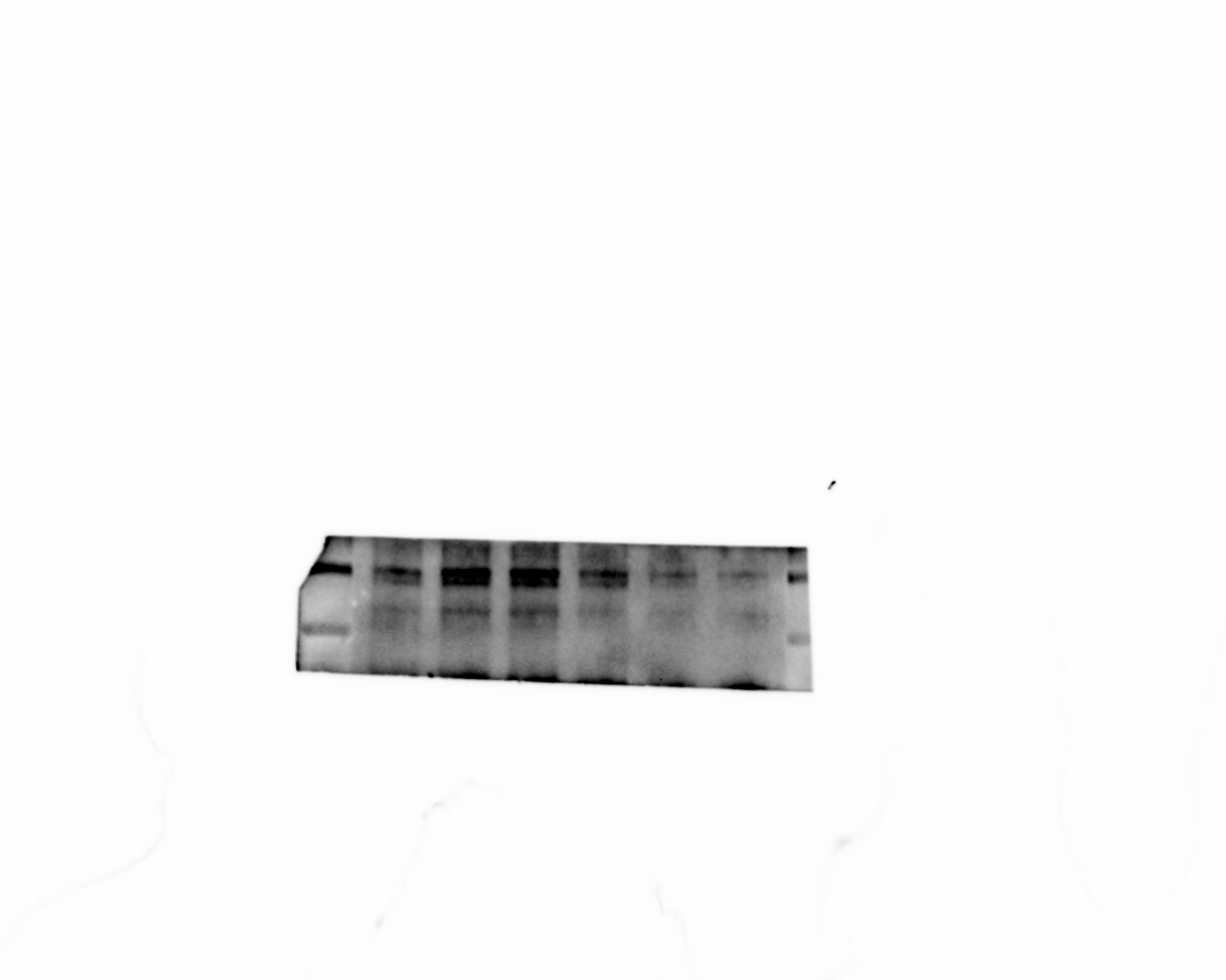


SHH


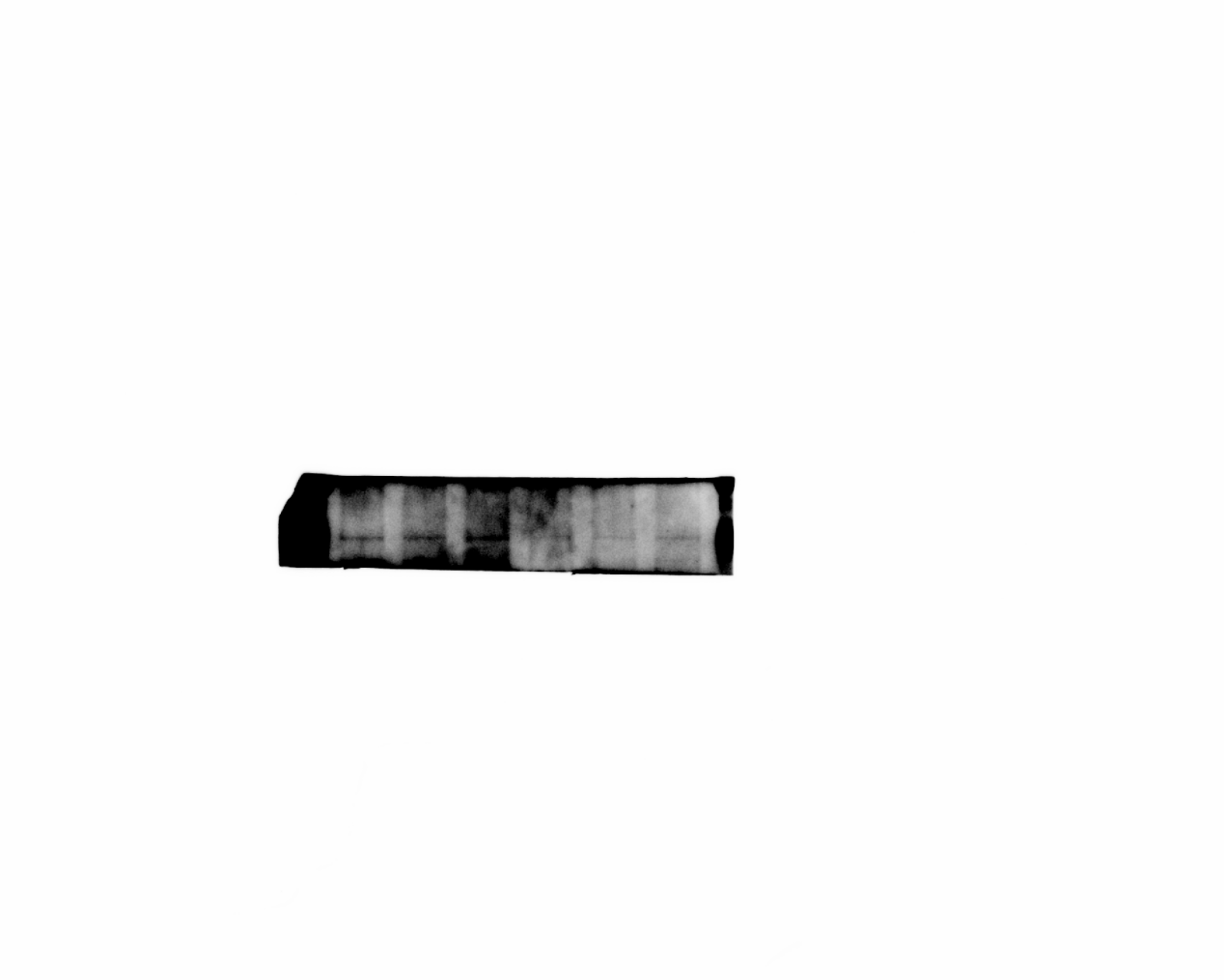


SMO


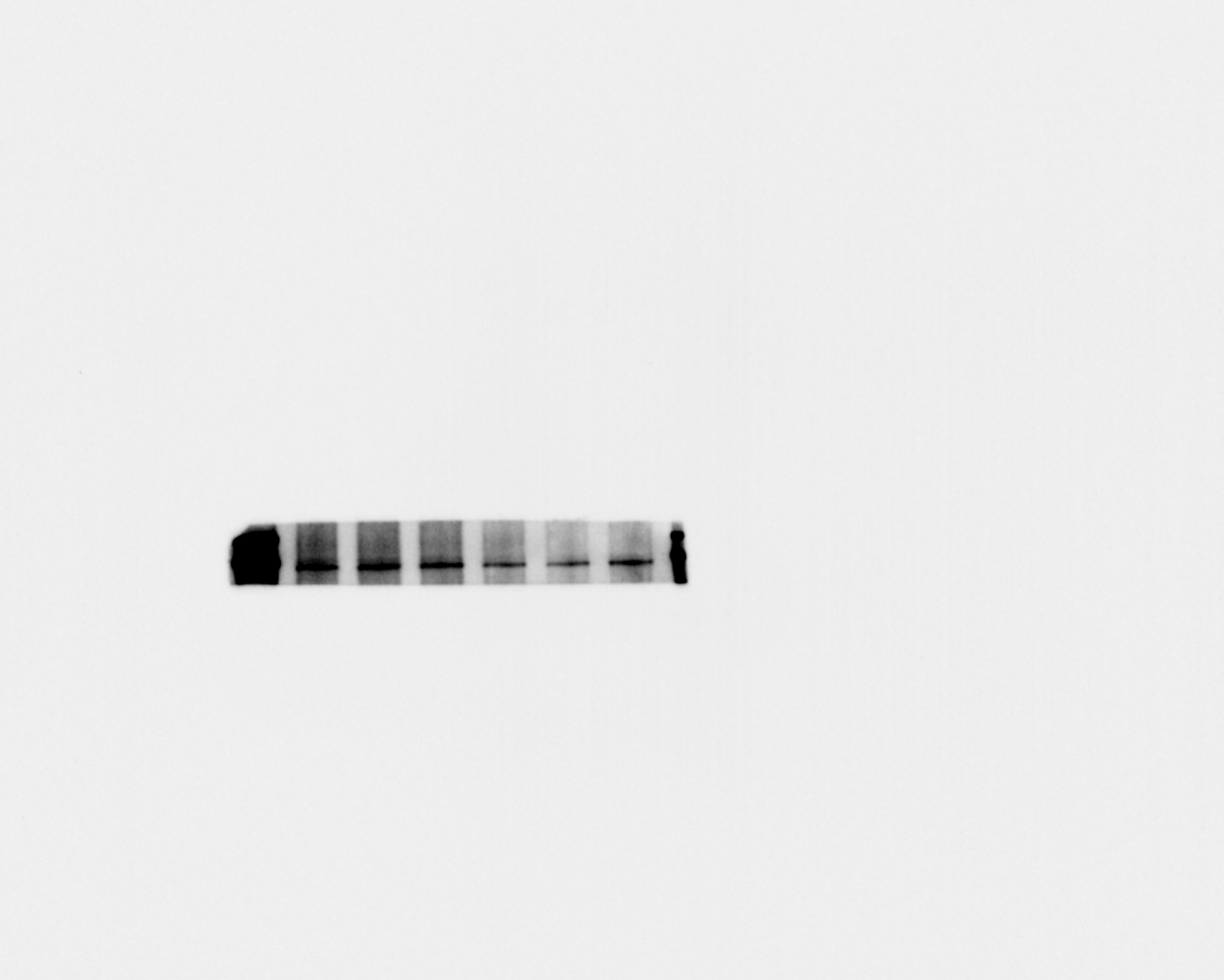


GLI1


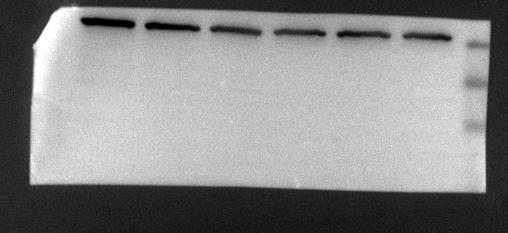


ACTIN

FIGURE 5 C


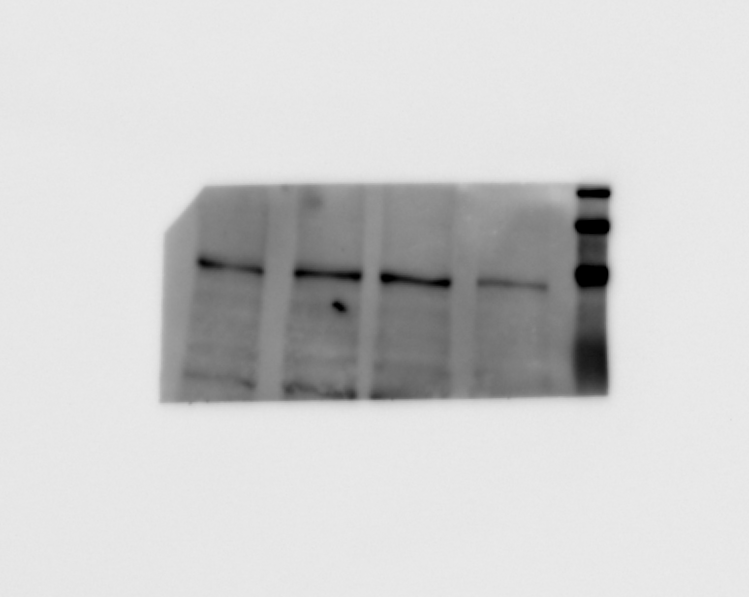


ERAP2


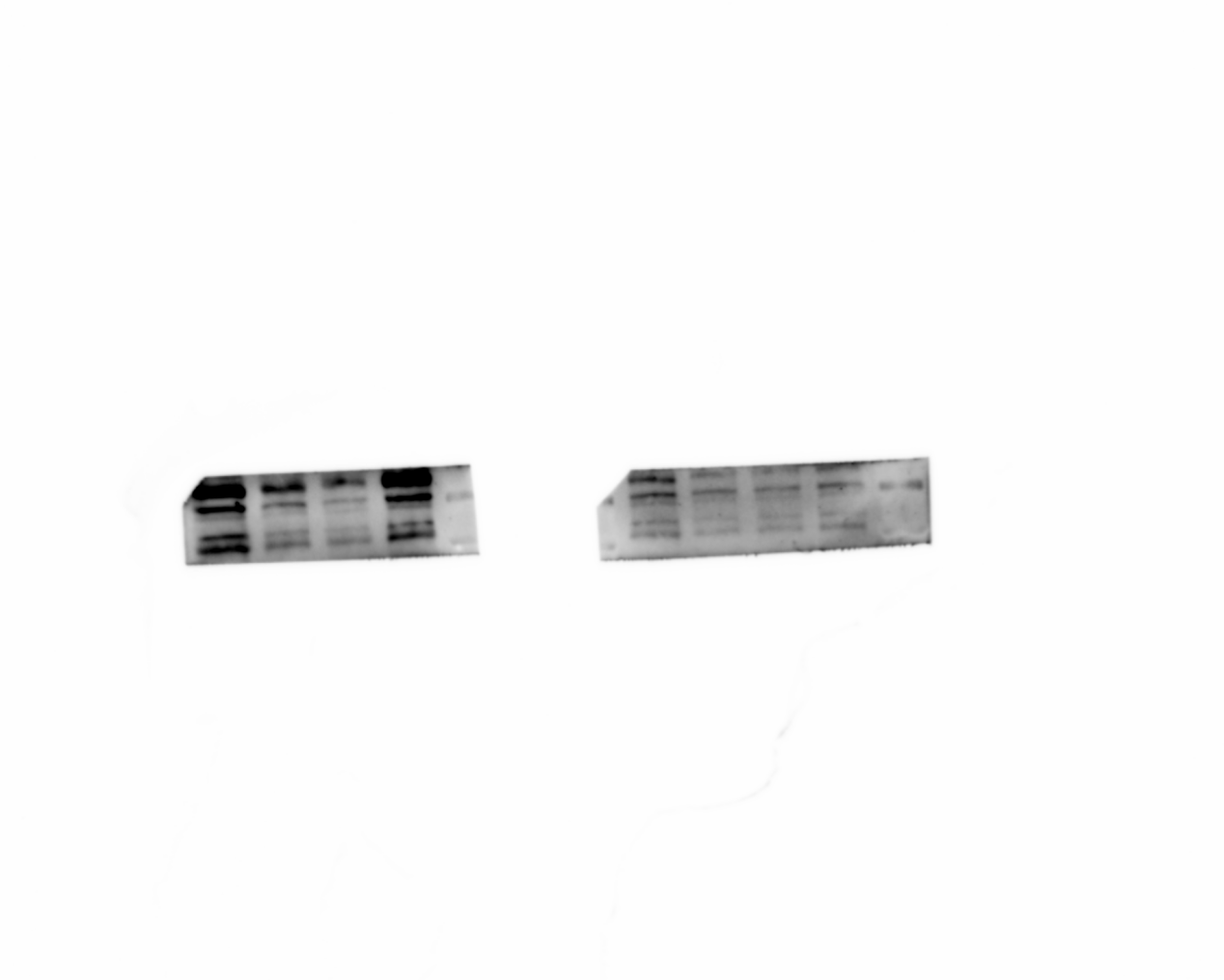


SHH


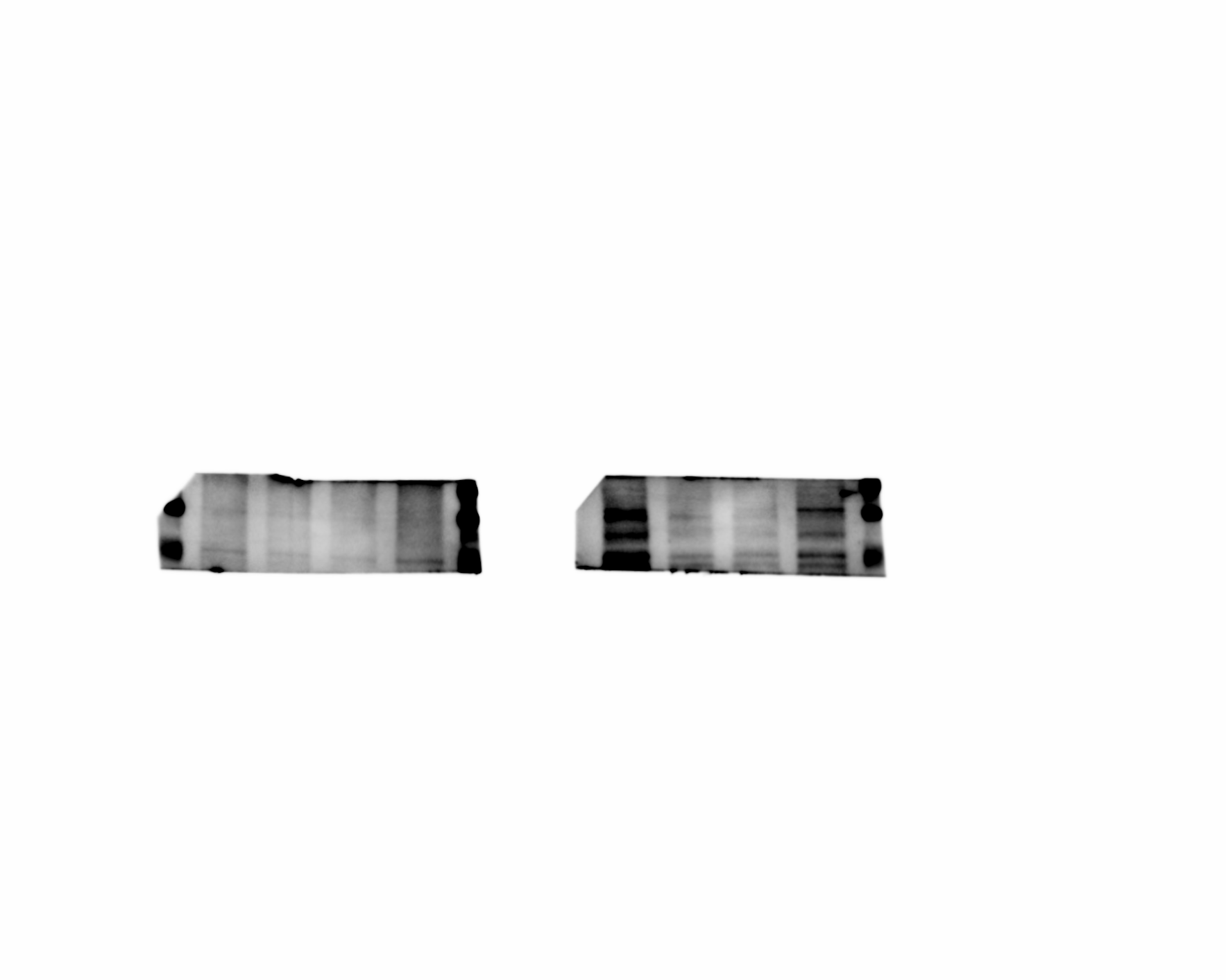


SMO


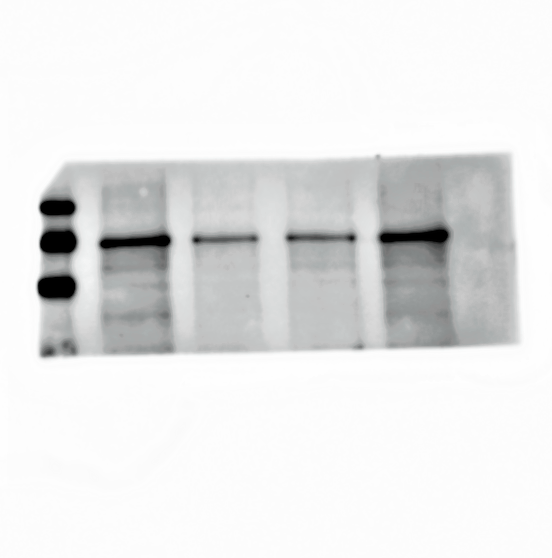


GLI1


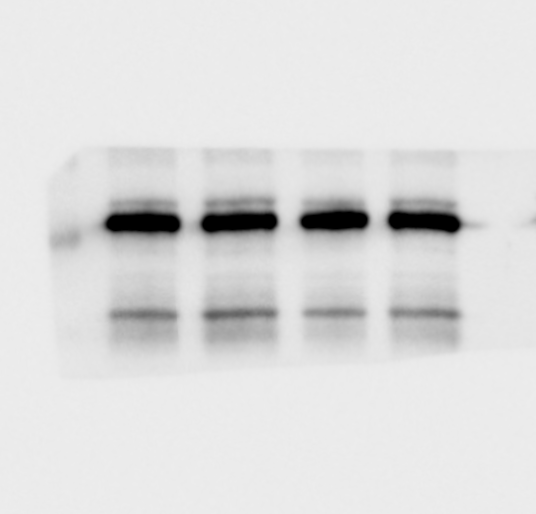


ACTIN

FIGURE 5 E


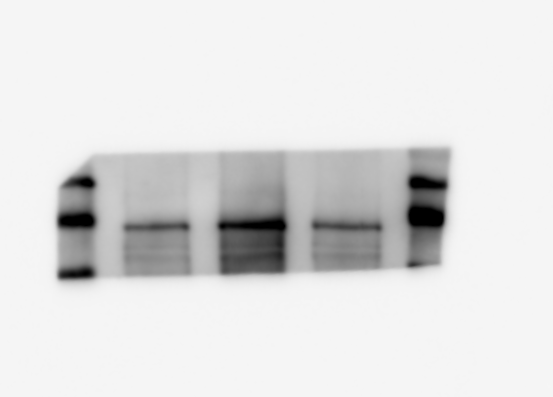


GLI1


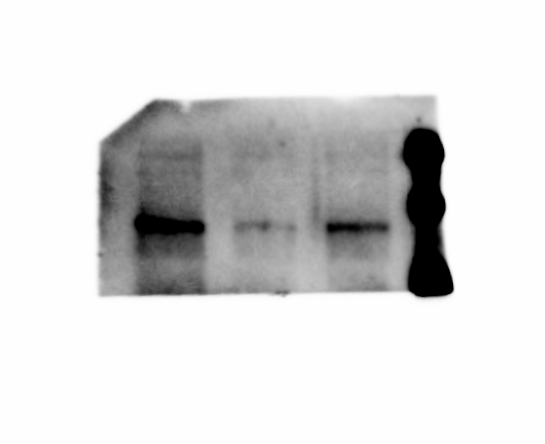


NLRP3


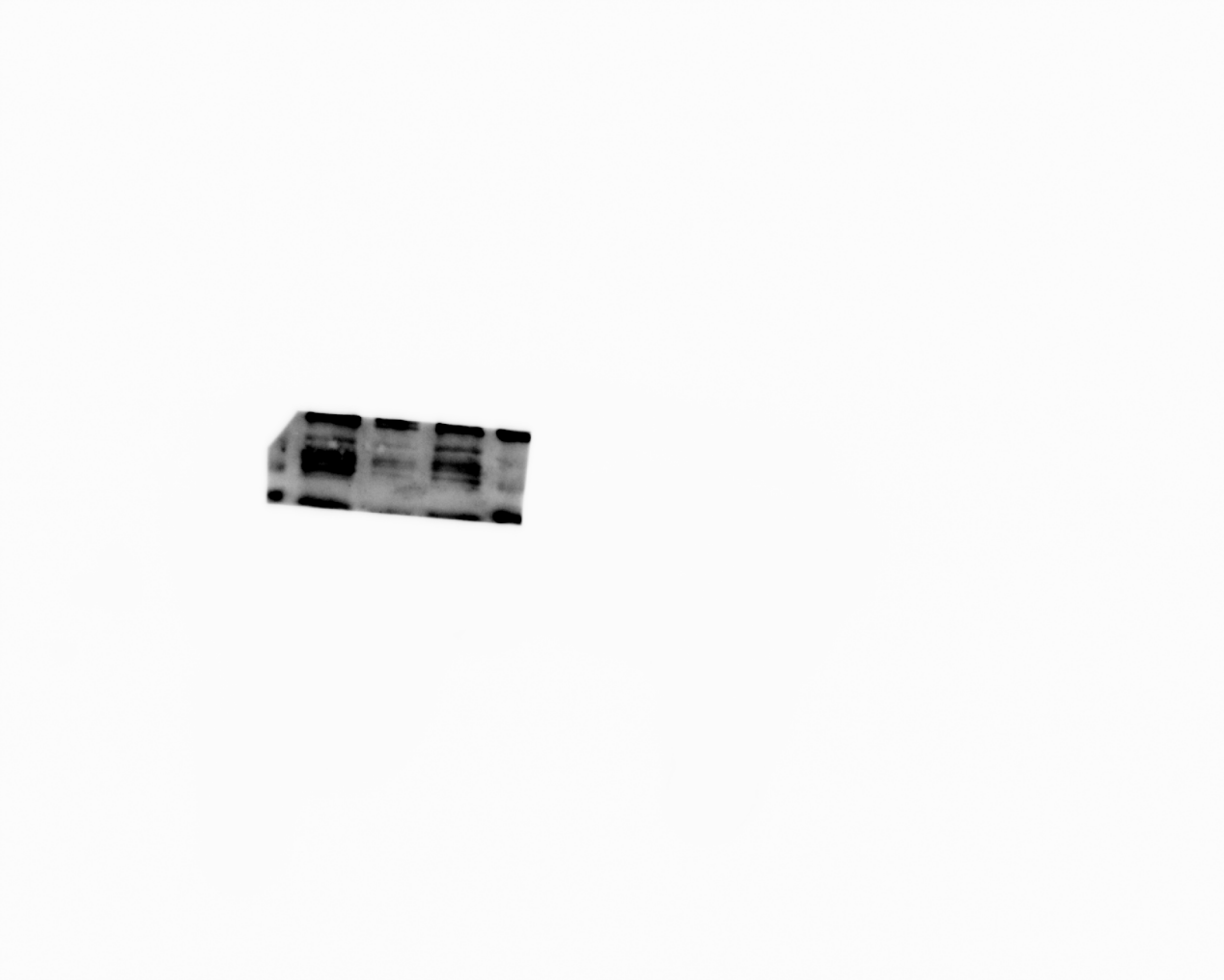


ASC


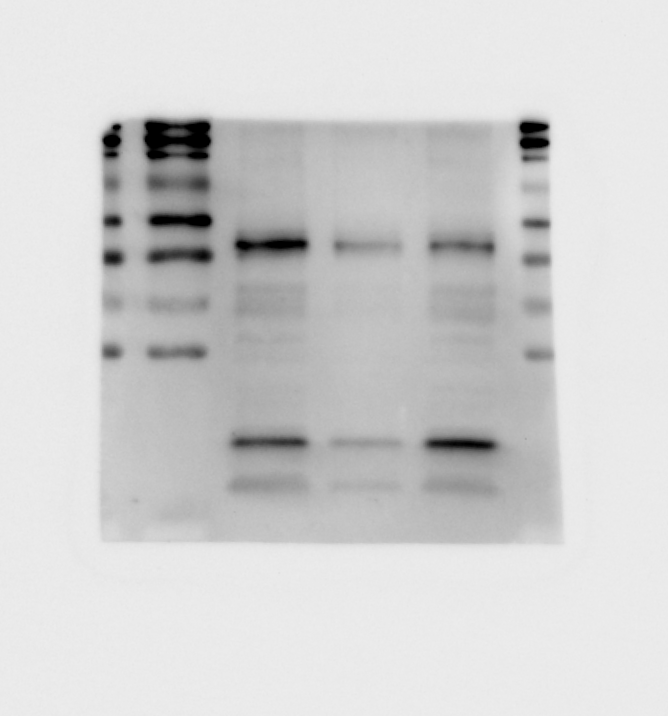


GASPASE-1


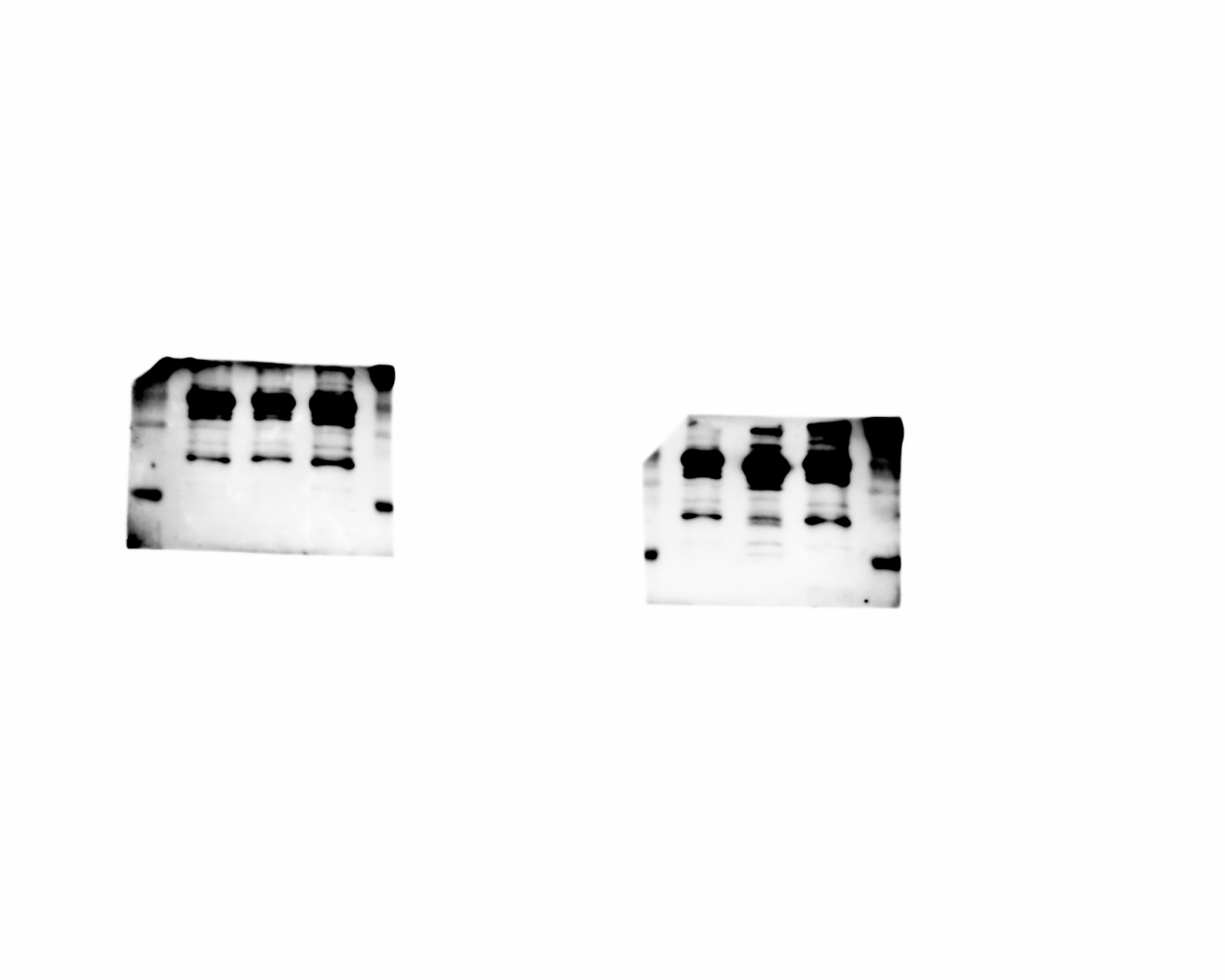


GSDMD


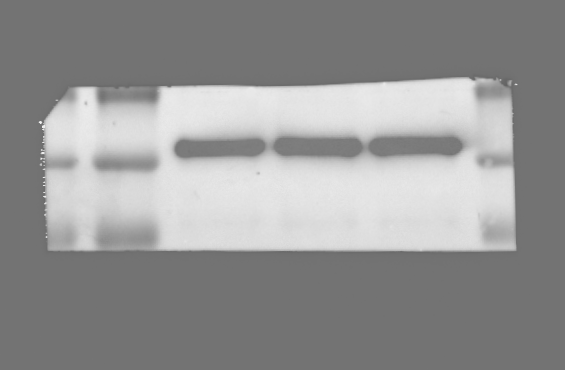


ACTIN


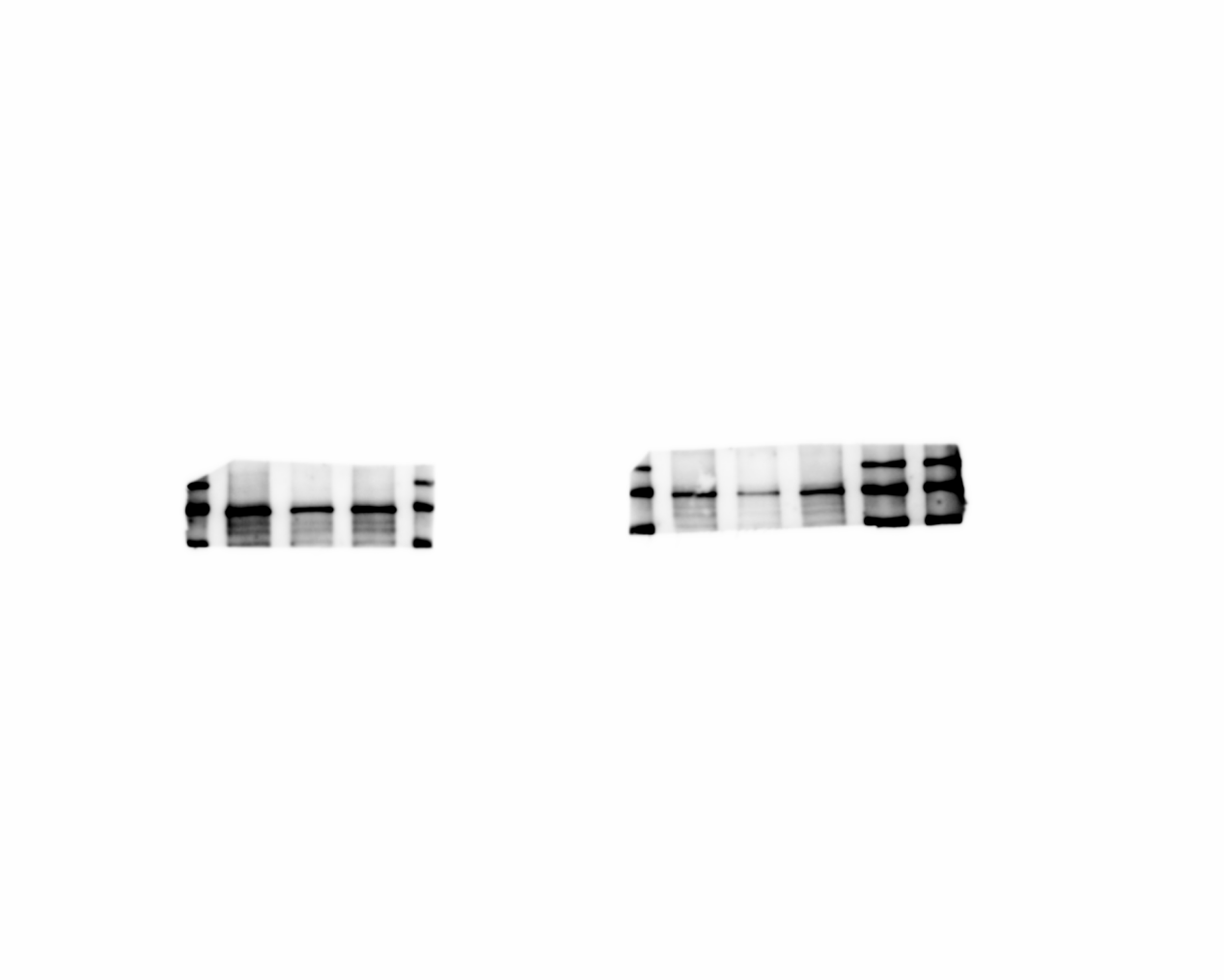
FIGURE 5 G

GLI1


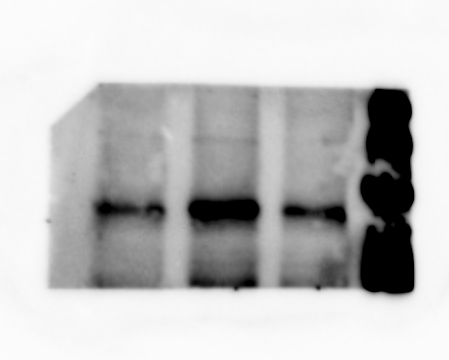


NLRP3


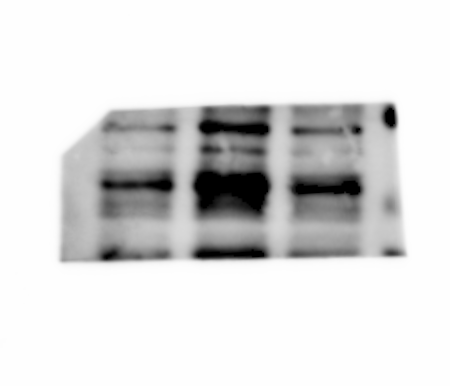


ASC


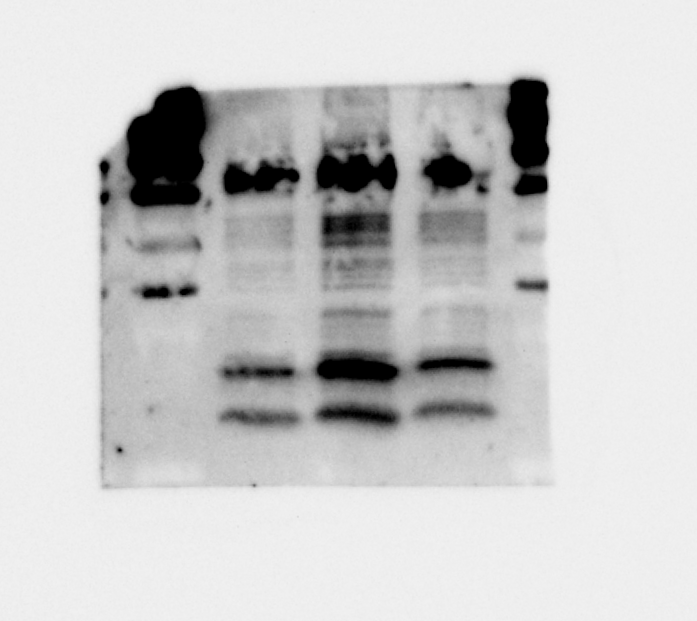


GASPASE-1


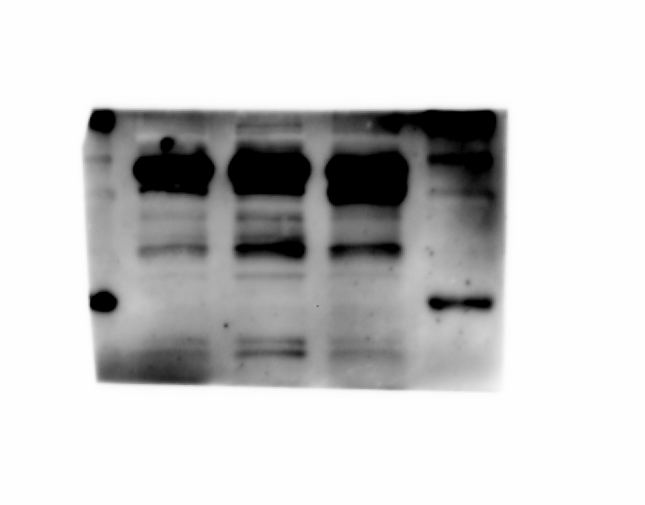


GSDMD


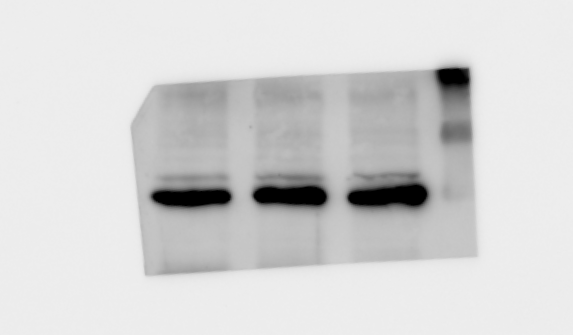


ACTIN

Supplemental Figure.2A


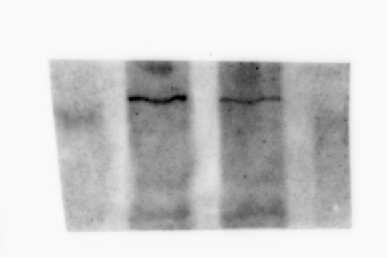


GLI1


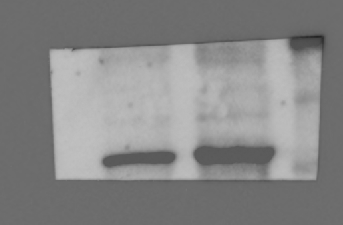


NLRP3


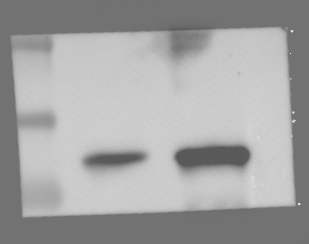


ASC


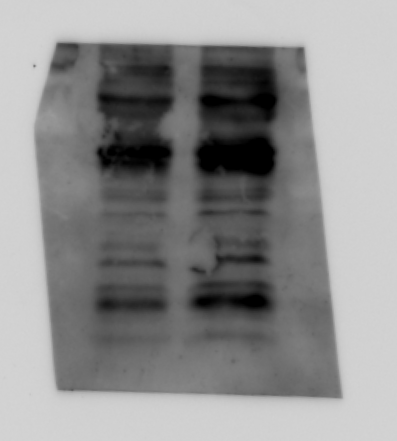


GASPASE-1


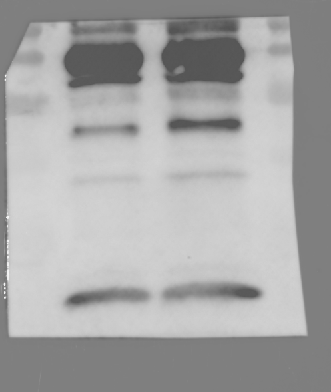


GSDMD


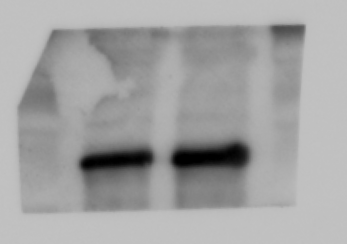


ACTIN

Supplemental Figure.2B


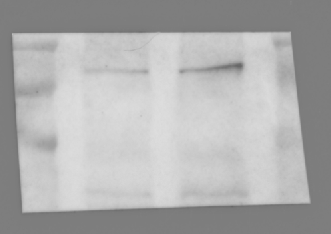


GLI1


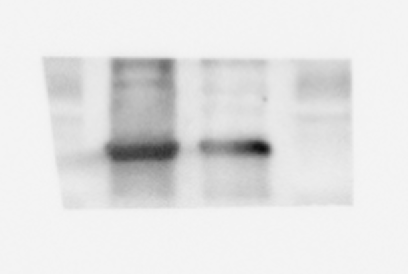


NLRP3


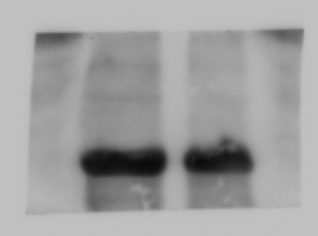


ASC


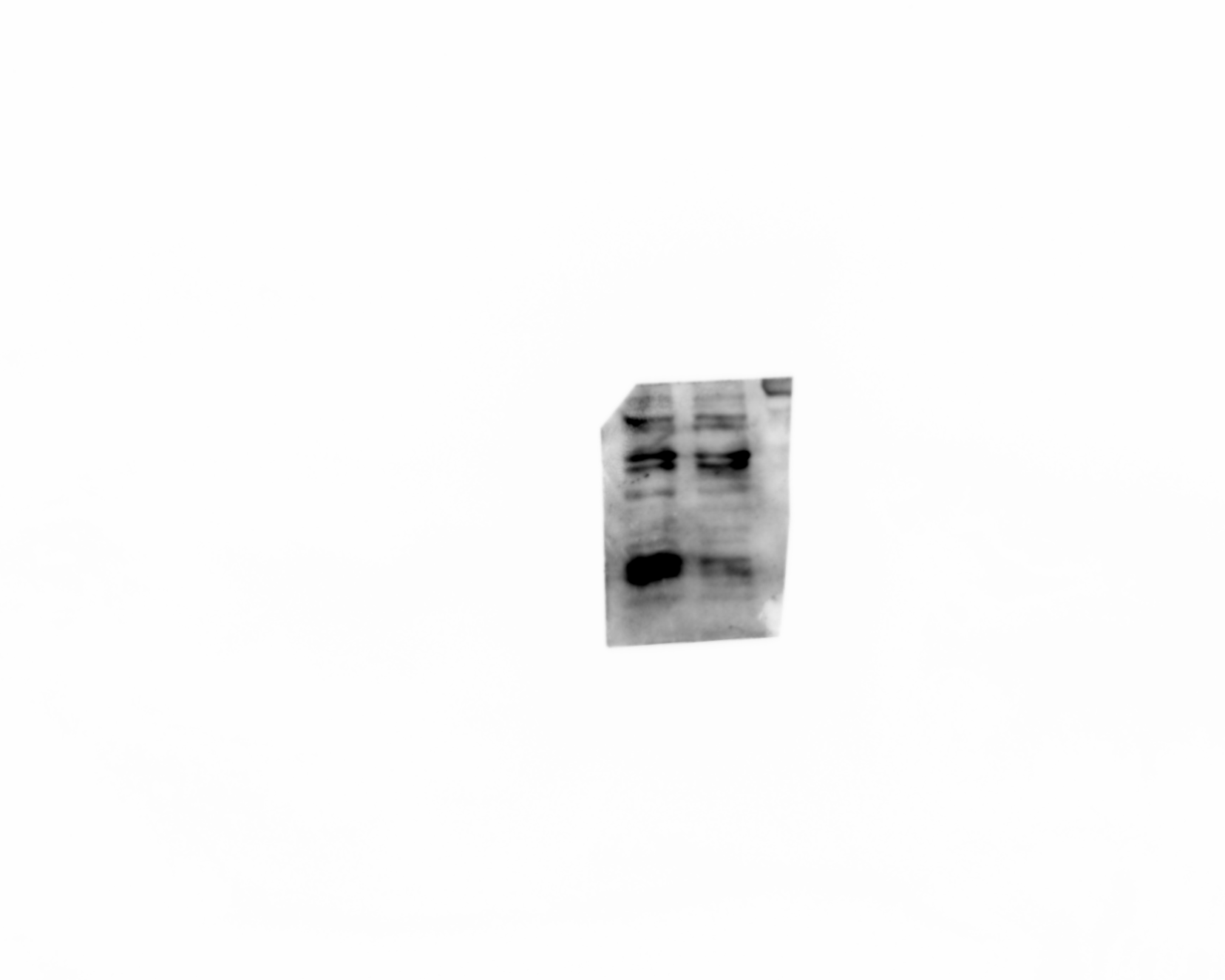


GASPASE-1


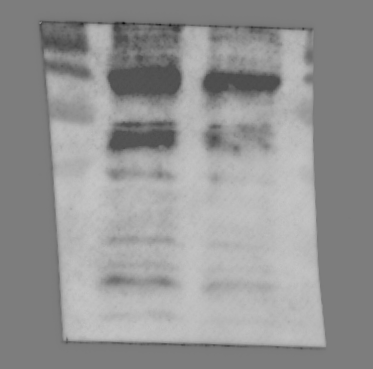


GSDMD


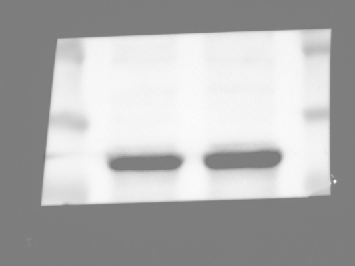
ACTIN
