# Supplementary material for: Endoplasmic reticulum aminopeptidase 2 regulates CD4+ T cells pyroptosis in rheumatoid arthritis
Source: Arthritis Res Ther. 2024 Jan 25;26:36. doi: 10.1186/s13075-024-03271-3 (PMC10810225; doi:10.1186/s13075-024-03271-3)
Supplement: Supplementary file 2 — Additional file 2: Supplemental Table 1. Primer sequences for RT-qPCR. Supplemental Figure 1. Comparative analysis of caspase activation in control and RA CD4+ T cells. Supplemental Figure 2. A, B ERAP2 induces pyroptosis in RA CD4+ T cells by inhibiting the Hedgehog signaling pathway. [file 13075_2024_3271_MOESM2_ESM.docx]

**Endoplasmic Reticulum Aminopeptidase 2 regulates CD4^+^ T cells pyroptosis in rheumatoid arthritis**

| Gene | Forward (5’-3’) | Reverse (5’-3’) |
| --- | --- | --- |
| β-actin | GTGCTATGTTGCTCTAGACTTCG | ATGCCACAGGATTCCATACC |
| ERAP2 | CACTAATGGGGAACGATTTCCTT | CTGACCAAGACTTCGATCTTCTC |
| ASC | GACGGGGCCAATACCACAC | TCTGTAACAAAAGTCGTGCTTCT |
| NLRP3 | TCTGTAACAAAAGTCGTGCTTCT | CCCGACAGTGGATATAGAACAGA |
| CASPASE-1 | GCTGAGGTTGACATCACAGGCA | TGCTGTCAGAGGTCTTGTGCTC |
| GSDMD | GTGTGTCAACCTGTCTATCAAGG | CATGGCATCGTAGAAGTGGAAG |
| TNFA | AAGGACACCATGAGCACTGAAAGC | AGGAAGGAGAAGAGGCTGAGGAAC |
| IL6 | GACAGCCACTCACCTCTTCAGAAC | GCCTCTTTGCTGCTTTCACACATG |
| IL1B | GCCAGTGAAATGATGGCTTATT | AGGAGCACTTCATCTGTTTAGG |
| IL10 | GCCGTGGAGCAGGTGAAGAATG | ATAGAGTCGCCACCCTGATGTCTC |
| TGFB1 | TATTGAGCACCTTGGGCACTGTTG | CCTTAACCTCTCTGGGCTTGTTTCC |

Supplemental Table.1 Primer sequences for RT-qPCR.

Supplemental Figure.1 Comparative analysis of caspase activation in control and RA CD4^+^ T cells.


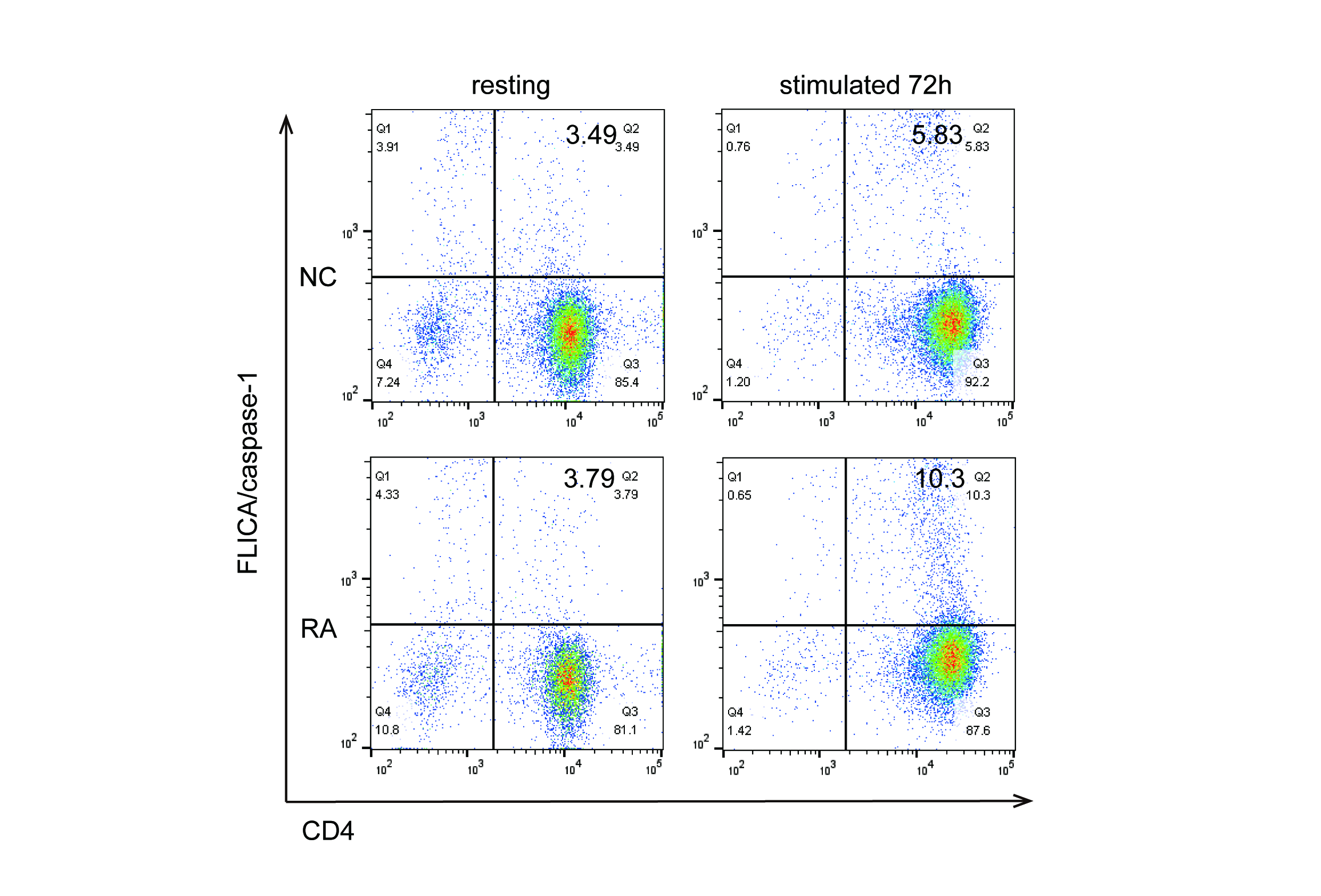


In the resting state, both RA and NC CD4^+^ T cells exhibited low levels of caspase-1 activation. However, as activation time passed, the difference in caspase-1 activation levels between the two groups became increasingly apparent. At 72 hours, a significant difference in active caspase-1 levels was observed between RA and NC.

Supplemental Figure.2A,B ERAP2 induces pyroptosis in RA CD4^+^ T cells by inhibiting the Hedgehog signaling pathway


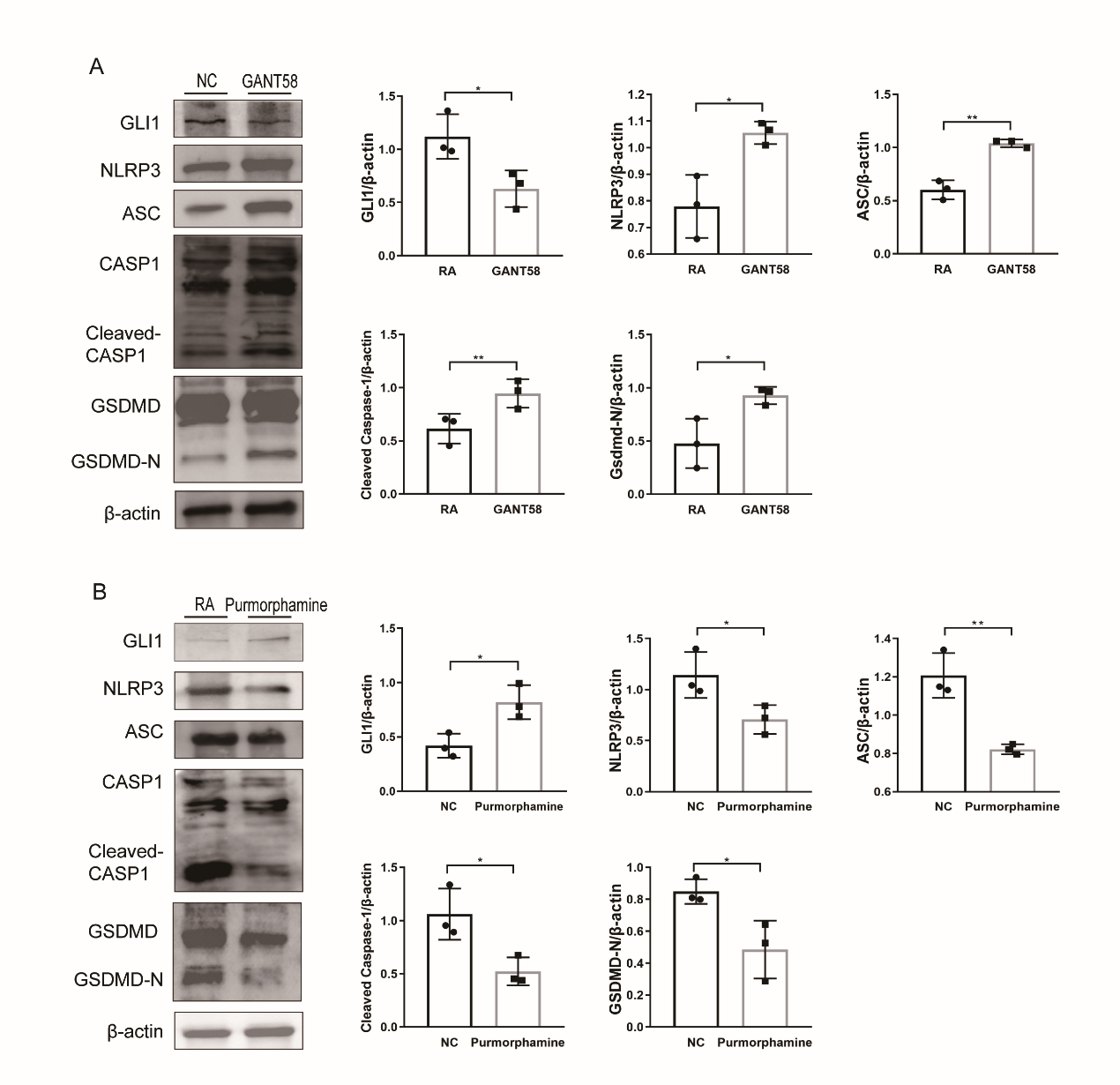


GLI1, NLRP3, ASC, cleaved caspase-1 and GSDMD-N expression in isolated CD4^+^ T cells with GANT58 treatment (20um) or purmorphamine (20um) is shown in representative immunoblots (left) and plots of the relative band density with normalization to β-actin expression (right).）

All data are presented as the mean±SD. Statistical significance was determined by Student’s t test; *p<0.05, **p<0.01, ***p<0.001, ****p<0.0001. NC, normal control; RA, rheumatoid arthritis.
